# Comparative mitochondrial genomics and phylogenetic relationships of the *Crossoptilon* species (Phasianidae, Galliformes)

**DOI:** 10.1186/s12864-015-1234-9

**Published:** 2015-02-05

**Authors:** Xuejuan Li, Yuan Huang, Fumin Lei

**Affiliations:** Co-Innovation Center for Qinba Regions’ Sustainable Development, School of Life Sciences, Shaanxi Normal University, Xi’an, 710062 China; Key Laboratory of the Zoological Systematics and Evolution, Institute of Zoology, the Chinese Academy of Sciences, Beijing, 100101 China

**Keywords:** Mitochondrial genome, *Crossoptilon*, Phylogeny, Divergence time, Ka/Ks

## Abstract

**Background:**

Phasianidae is a family of Galliformes containing 38 genera and approximately 138 species, which is grouped into two tribes based on their morphological features, the Pheasants and Partridges. Several studies have attempted to reconstruct the phylogenetic relationships of the Phasianidae, but many questions still remain unaddressed, such as the taxonomic status and phylogenetic relationships among *Crossoptilon* species. The mitochondrial genome (mitogenome) has been extensively used to infer avian genetic diversification with reasonable resolution. Here, we sequenced the entire mitogenomes of three *Crossoptilon* species (*C. harmani*, *C. mantchuricum* and *C. crossoptilon*) to investigate their evolutionary relationship among *Crossoptilon* species.

**Results:**

The complete mitogenomes of *C. harmani*, *C. mantchuricum* and *C. crossoptilon* are 16682 bp, 16690 bp and 16680 bp in length, respectively, encoding a standard set of 13 protein-coding genes, 2 ribosomal RNA genes, 22 transfer RNA genes, and a putative control region. *C. auritum* and *C. mantchuricum* are more closely related genetically, whereas *C. harmani* is more closely related to *C. crossoptilon. Crossoptilon* has a closer relationship with *Lophura*, and the following phylogenetic relationship was reconstructed: ((*Crossoptilon* + *Lophura*) + (*Phasianus* + *Chrysolophus*)). The divergence time between the clades *C. harmani*-*C. crossoptilon* and *C. mantchuricum*-*C. auritum* is consistent with the uplift of the Tibetan Plateau during the Tertiary Pliocene. The Ka/Ks analysis showed that *atp8* gene in the *Crossoptilon* likely experienced a strong selective pressure in adaptation to the plateau environment.

**Conclusions:**

*C. auritum* with *C. mantchuricum* and *C. harmani* with *C. crossoptilon* form two pairs of sister groups. The genetic distance between *C. harmani* and *C. crossoptilon* is far less than the interspecific distance and is close to the intraspecific distance of *Crossoptilon*, indicating that *C. harmani* is much more closely related to *C. crossoptilon*. Our mito-phylogenomic analysis supports the monophyly of *Crossoptilon* and its closer relationship with *Lophura*. The uplift of Tibetan Plateau is suggested to impact the divergence between *C. harmani*-*C. crossoptilon* clade and *C. mantchuricum*-*C. auritum* clade during the Tertiary Pliocene. *Atp8* gene in the *Crossoptilon* species might have experienced a strong selective pressure for adaptation to the plateau environment.

**Electronic supplementary material:**

The online version of this article (doi:10.1186/s12864-015-1234-9) contains supplementary material, which is available to authorized users.

## Background

*Crossoptilon*, belonging to Phasianidae in Galliformes, is a rare but important genus endemic to China. The four previous recognised *Crossoptilon* species are *C. harmani*, *C. mantchuricum*, *C. crossoptilon* and *C. auritum* [1,2]. *C. harmani* is only distributed in a typical alpine and taiga habitats at elevations of 3500–3900 meters of southeastern Tibet. *C. mantchuricum* is mainly distributed in Lvliang Mountains of Shanxi Prov., Xiaowutai Mountains of Hebei Prov., Dongling Mountains of Beijing and some other local areas [1]. *C. crossoptilon* is only present in western Sichuan Prov., northwestern Yunnan Prov., southeastern Qinghai Prov. and eastern Tibet at high mountain areas [3,4]. It is common at high altitudes (3000–4300 m) and exists in coniferous forests, mixed broadleaf-conifer forests and alpine scrubs [4]. *C. auritum* is only encountered in the mountainous regions of Qinghai, Gansu and Sichuan provinces and Ningxia Hui Autonomous Region [1].

The taxonomic status and phylogenetic position of *C. harmani* are controversial [4-10]. Whereas some studies have proposed that it is an independent species [1,2,6,11], others have suggested that it is a subspecies of *C. crossoptilon* [4,5,12-14]. The *C. mantchuricum* taxon is believed to have diverged first, and *C. crossoptilon* and *C. auritum* are considered more closely related than the other species [7,9,10]. Other studies have suggested that *C. crossoptilon* is a relatively original species and that *C. auritum* and *C. mantchuricum* are more closely related genetically [1,4-6,8,12,14].

Previous studies on *Crossoptilon* species have focused on their distribution, ecology and behaviour. Some studies have used single gene or partial sequences in reconstructing their phylogenetic relationships [15], but a comprehensive study of the entire mitogenomes has rarely been attempted. To reconstruct the phylogenetic relationships among *Crossoptilon* and other Phasianidae species, the entire mitogenomes of *C. harmani*, *C. mantchuricum* and *C. crossoptilon* were sequenced, and thus four species of the genus *Crossoptilon* were thoroughly compared. Furthermore, the estimated divergence times of the four *Crossoptilon* species were calculated, and a Ka/Ks analysis was used to estimate the adaptation of the *Crossoptilon* mitogenome to different environments.

## Methods

### Sample collection and DNA extraction

Samples of *C. harmani* were collected from the Nagqu area in Tibet, China, and voucher specimen was deposited in Institute of Zoology, Shaanxi Normal University; *C. mantchuricum* and *C. crossoptilon* were collected from the Beijing Zoo, and samples were obtained from bird specimen collections of the National Zoological Museum, Institute of Zoology, Chinese Academy of Sciences. The collection was under the permit from Forestry Department and conformed to the National Wildlife Conservation Law in China. No living animal experiments were conducted in the current research. All the samples were preserved in 100% ethanol and stored at −20°C. The total genomic DNA was extracted from the liver/muscle tissue using the standard phenol/chloroform method [16].

### PCR amplification and sequencing

The PCRs were performed under the following conditions, 2 min initial denaturation at 93°C, 40 cycles of: 10 s denaturation at 92°C, 30 s annealing at 58–53°C, 10 min elongation at 68°C in the preliminary 20 cycles, and 10 s denaturation at 92°C, 30 s annealing at 53°C, and 10 min elongation at 68°C with 20 s per cycle added to the elongation step in the succeeding 20 cycles, and finally an extension for 7 min at 68°C. The amplifications were performed in 15 μL reactions containing 2.4 μL of 2.5 mM dNTPs, 2.1 μL of each primer at 10 μΜ, 1.5 μl of 10× LA PCR Buffer I (Mg^2+^-free), 1.5 μL of 25 mM MgCl_2_, 1 μL of DNA template, 0.18 μL of 5 U/μl LA Taq polymerase (Takara, Dalian, China) and 4.22 μL ddH_2_O.

The sequences of the primers used for PCR amplification and sequencing of the mitochondrial genes were obtained from Sorenson (2003) [17] with minor changes (Additional file 1). The mitogenome of *C. harmani* was amplified in seven parts and the gaps were bridged using other adjacent primers. The PCR products were purified using the DNA Agarose Gel Extraction Kit (Bioteke, Beijing, China) after separation by electrophoresis on a 0.8% agarose gel. After separation and purification, the PCR products were sequenced by Sangon Biotech (Shanghai) Co., LTD. using the Primer-Walking method. The *C. mantchuricum* and *C. crossoptilon* mitogenomes were amplified in 12 or 13 fragments, and sequencing was performed using the Illumina Hiseq2000 high-throughput sequencing system of Shenzhen Huada Gene Technology Co., LTD. The gaps in the assembly after high-throughput sequencing were filled in by direct sequencing using the ABI 3730 DNA sequencer by Sangon Biotech (Shanghai) Co., LTD., using adjacent PCR primers.

### Gene identification and genome analyses

The Staden sequence analysis package [18] was used for sequence assembly and annotation of *C. harmani* mitogenome. The complete genome assemblies for the mitogenomes of *C. mantchuricum* and *C. crossoptilon* were performed using the SOAP de novo software. Most tRNA genes were identified using tRNAscan-SE 1.21 [19] under the ‘tRNAscan only’ search mode, with the vertebrate mitochondrial genetic code and ‘mito/chloroplast’ source. The protein-coding genes (PCGs), rRNA genes and the remaining putative tRNA genes that were not identified by tRNAscan-SE were identified by sequence comparison with other Galliformes species. The *rrnS* secondary structure of *Crossoptilon* was predicted based on the structure of *Gallus gallus* and *Anas platyrhynchos* obtained from the Comparative RNA Web (CRW) [20], and *Pseudopodoces humilis* (now as *Parus humilis*) structure [16]. The *rrnL* secondary structure was predicted based on the structure of *Xenopus laevis* obtained from the CRW database, *Bos taurus* [21] and *P. humilis* structures [16]. The RNAstructure software was used to identify and draw potential secondary structures in the single-stranded control region. The nucleotide compositions of the mitogenomes and amino acid information were analysed using MEGA 4.1 [22].

### Sequence alignments

Along with the entire mitogenomes obtained in this study, 42 Galliformes sequences were used in the phylogenetic analysis, including two outgroups (*Numida meleagris* and *Alectura lathami*). The DNA sequences of the other species used in the phylogenetic analyses were downloaded from GenBank (the accession numbers and key information are shown in Additional file 2). The tRNA and rRNA genes and the CR were individually aligned using ClustalX 1.83 [23] with the default settings. All 13 protein-coding genes were translated into amino acids, and then aligned using MEGA 4.1 [22] with default parameters for each gene, and finally retranslated into nucleotide sequences.

### Phylogenetic analyses of Phasianidae

Datasets containing 13 protein-coding genes (PCG) and all 37 genes plus the control region (mitogenome) were used to study the phylogenetic relationships within Phasianidae. Phylogenetic analysis based on nucleotide sequences was performed using PAUP*4.0b10 for the Maximum parsimony (MP) method [24], RAxML-7.0.3 for maximum likelihood (ML) [25] and MrBayes 3.1.2 for Bayesian inference (BI) [26]. For ML and BI analyses, models of the concatenated nucleotide sequences datasets were assessed independently using AICc in MrModeltest2.2 [27]. The best fit model GTR + I + G was chosen for the likelihood and Bayesian analyses. A consensus tree was generated for MP analysis under the majority rule. The reliability of the clades in the phylogenetic trees was assessed by bootstrap probabilities (BSP) computed using 1000 replicates, with random addition for each bootstrap replicate. The 1000 replicates bootstrap support was also performed in the ML analysis. Bayesian analysis with Markov Chain Monte Carlo sampling was run for 1000000 generations saving a tree every 100 generations, with one cold and three heated chains, and the burn-in time was determined by the time to convergence of the likelihood scores. The Bayesian posterior probabilities (BPP) were estimated on a 50% majority rule consensus tree of the remaining trees.

We examined the performance of individual genes and datasets based on nucleotides; PBS analyses were performed in the program combining TreeRot.v3 [28] and PAUP*4.0b10 [24]. The following datasets were used for analysis: 13 protein-coding genes, *rrnS*, *rrnL*, CR partitions, the first, second and third codons of PCG, the three tRNA gene cluster (IQM, WANCY and HSL), ATP (*atp6* + *atp8*), COX (*cox1* + *cox2* + *cox3*) and NADH (*nad1* + *nad2* + *nad3* + *nad4* + *nad4L* + *nad5* + *nad6*).

The MEGA 4.1 [22] was used to calculate the pairwise genetic distance for four *Crossoptilon* species with default parameters. The mitogenome aligned data and four single genes (*nad2*, CR, *cytb* and *rrnS*) obtained from GenBank (Additional file 3) were used to calculate the genetic distances. These genes were aligned singly, and be adjusted to consistent sequence lengths manually.

### Divergence time estimates focused on *Crossoptilon*

Along with the PCG dataset obtained in this study, 42 Galliformes sequences were used to estimate the divergence time of the *Crossoptilon* species. The divergence time of *Crossoptilon* species was calculated using the Bayesian procedure implemented in BEAST v. 1.7.2 [29,30]. A relaxed clock was used with rates complying with a log-normal distribution [31]. The GTR + I + G model and a Yule prior were used in the analysis. The calibration points were based on the fossil records showing that stem Numididae-Phasianidae split at 50–54 Mya (million years ago) [32]; *Arborophila rufipectus* diverged from the other lineages in the Galliformes around 39 Mya [33]; *Coturnix*-*Gallus* split at 35 Mya [34-36]. The results of runs of 10 million generations were used after a burn-in of 100.

### Ka and Ks analysis

To better understand the evolution at the DNA level and the role of selection in the four *Crossoptilon* species, we calculated the nonsynonymous and synonymous substitution rates using Kaks_calculator 2.0 [37] for six groups [*C. harmani*-*C. mantchuricum* (*C.har*-*C.man*), *C. mantchuricum*-*C. crossoptilon* (*C.man*-*C.cro*), *C. harman*i-*C. crossoptilon* (*C.har*-*C.cro*), *C. harmani*-*C. auritum* (*C.har*-*C.aur*), *C. mantchuricum*-*C. auritum* (*C.man*-*C.aur*), and *C. crossoptilon*-*C. auritum* (*C.cro*-*C.aur*)]. The ratio of nonsynonymous substitution rate (Ka) to synonymous substitution rate (Ks) is widely used as an indicator of selective pressure at the sequence level among different species. It is commonly accepted that Ka > Ks, Ka = Ks, and Ka < Ks generally indicate positive selection, neutral mutation, and negative selection, respectively [38,39]. To calculate Ka, Ks and Ka/Ks, a model averaging method was selected. This method includes 14 different models for calculation and derived the average values for Ka, Ks, and Ka/Ks [37]. The genetic code selected was the ‘vertebrate mitochondrial code’. To further study the selective pressure acted on each protein-coding gene in the genus *Crossoptilon*, CodeML in PAMLX software [40] was used to find sites under strong selective pressure. The secondary structure analysis of amino acid was performed by using an online software TOPCONS [41].

## Results

Comparison of the two sequencing methods used in this study revealed that high-throughput sequencing has greater coverage and accuracy relative to standard sequencing, albeit with higher cost. Using standard sequencing, we obtained 62 effective sequences with 2.63-fold coverage. In contrast, the high-throughput sequencing yielded effective assembly data with sequence depths (X) of 7604.35 for *C. mantchuricum* and 7810.82 for *C. crossoptilon* after filtering out some reads, such as low-quality or adapter-sequence-polluted reads.

The complete mitogenomes of *C. harmani*, *C. mantchuricum* and *C. crossoptilon* are 16682 bp, 16690 bp and 16680 bp in length, respectively. The annotated genomes have been deposited in the GenBank database (accession numbers: KP259806-KP259808). The mitochondrial genes of three *Crossoptilon* species are coded on the H-strand, except for one protein-coding gene (*nad6*) and eight tRNA genes (*trnQ*, *trnA*, *trnN*, *trnC*, *trnY*, *trnS (UCN)*, *trnP*, and *trnE*) (Table 1). Consistent with previous results on the Phasianidae mitogenomes, the mitochondrial sequences of four *Crossoptilon* species (*C. harmani*, *C. mantchuricum*, *C. crossoptilon* and *C. auritum*) are biased toward adenine and thymine (54.3%, 54.2%, 54.3%, and 54.2%, respectively). The GC skew in *Crossoptilon* mitogenomes is similar, with parts of the CR and *rrnS* containing significantly higher skews than other regions. The CR, *trnF*, *rrnS*, *trnV*, *rrnL* and *cox1* regions have strong GC skews.

**Table 1:** **Localisations and features of genes in the mitogenomes of three** ***Crossoptilon*** **species**

**Gene**	**Coding strand**	***C. harmani***			***C. mantchuricum***			***C. crossoptilon***		
		**From**	**To**	**Initiator/terminator**	**From**	**To**	**Initiator/terminator**	**From**	**To**	**Initiator/terminator**
CR		1	1146		1	1146		1	1146	
*trnF*	J	1147	1214		1147	1214		1147	1214	
*rrnS*	J	1214	2178		1214	2179		1214	2178	
*trnV*	J	2179	2251		2180	2252		2179	2251	
*rrnL*	J	2252	3861		2253	3869		2252	3859	
*trnL(UUR)*	J	3862	3935		3870	3943		3860	3933	
*nad1*	J	3947	4921	ATG/TAA	3955	4929	ATG/TAA	3945	4919	ATG/TAA
*trnI*	J	4922	4993		4930	5001		4920	4991	
*trnQ*	N	5000	5070		5008	5078		4998	5068	
*trnM*	J	5070	5138		5078	5146		5068	5136	
*nad2*	J	5139	6177	ATG/T	5147	6185	ATG/T	5137	6175	ATG/T
*trnW*	J	6178	6255		6186	6263		6176	6253	
*trnA*	N	6262	6330		6270	6338		6260	6328	
*trnN*	N	6334	6406		6342	6414		6332	6404	
*trnC*	N	6409	6474		6417	6482		6407	6472	
*trnY*	N	6474	6544		6482	6552		6472	6542	
*cox1*	J	6546	8096	GTG/AGG	6554	8104	GTG/AGG	6544	8094	GTG/AGG
*trnS(UCN)*	N	8088	8162		8096	8170		8086	8160	
*trnD*	J	8165	8233		8173	8241		8163	8231	
*cox2*	J	8235	8918	ATG/TAA	8243	8926	ATG/TAA	8233	8916	ATG/TAA
*trnK*	J	8920	8990		8928	8997		8918	8988	
*atp8*	J	8992	9156	ATG/TAA	8999	9163	ATG/TAA	8990	9154	ATG/TAA
*atp6*	J	9147	9830	ATG/TAA	9154	9837	ATG/TAA	9145	9828	ATG/TAA
*cox3*	J	9830	10613	ATG/T	9837	10620	ATG/T	9828	10611	ATG/T
*trnG*	J	10614	10682		10621	10689		10612	10680	
*nad3*	J	10683	11034	ATG/TAA	10690	11041	ATG/TAA	10681	11032	ATG/TAA
*trnR*	J	11036	11104		11043	11111		11034	11102	
*nad4L*	J	11105	11401	ATG/TAA	11112	11408	ATG/TAA	11103	11399	ATG/TAA
*nad4*	J	11395	12772	ATG/T	11402	12779	ATG/T	11393	12770	ATG/T
*trnH*	J	12773	12841		12780	12848		12771	12839	
*trnS(AGY)*	J	12842	12907		12849	12914		12840	12905	
*trnL(CUN)*	J	12908	12978		12915	12985		12906	12976	
*nad5*	J	12979	14796	ATG/TAA	12986	14803	GTG/TAA	12977	14794	ATG/TAA
*cytb*	J	14803	15945	ATG/TAG	14810	15952	ATG/TAG	14801	15943	ATG/TAG
*trnT*	J	15947	16015		15954	16022		15945	16013	
*trnP*	N	16018	16086		16025	16093		16016	16084	
*nad6*	N	16092	16613	ATG/AGG	16100	16621	ATG/TAG	16090	16611	ATG/AGG
*trnE*	N	16615	16682		16623	16690		16613	16680	

### Protein-coding genes

The 13 protein-coding genes of *Crossoptilon* genomes are similar to most other Phasianidae species, with *nad5* and *atp8* being the longest and shortest genes, respectively. The total length of the PCGs in each *Crossoptilon* species is 11358 bp after removing termination codons, containing approximately 3786 codons. The A + T content of the 13 PCGs is 53.4% in *C. harmani* and *C. crossoptilon*, and 53.3% in *C. auritum* and *C. mantchuricum*. Analysis of the base composition at each codon position of the concatenated PCGs shows that the second codon position has a higher A + T content (57.9% in *C. auritum* and *C. mantchuricum*, 58.1% in *C. harmani*, and 58.0% in *C. crossoptilon*, respectively) than the first and third codon positions. The amino acid frequencies in the *Crossoptilon* PCGs are similar, with Leu significantly more frequent than other amino acids. The different codon positions have the same base distributions, with C and G as the most and least frequent bases in the third codon, respectively.

All PCGs in *Crossoptilon* species start with the typical ATG codon, except the *nad5* gene in *C. mantchuricum* and the *cox1* gene in four *Crossoptilon* species, which start with GTG. Four types of stop codons are used by the coding genes, including TAA and TAG for most genes, AGG for *cox1* in *Crossoptilon* and *nad6* in *C. harmani* and *C. crossoptilon*, and an incomplete stop codon T- for *cox3*, *nad4* and *nad2* in four *Crossoptilon* species, respectively.

### RNA genes

Similar to previously sequenced mitogenomes, the genomes sequenced in this study contain 2 rRNA genes encoding the small and large rRNA subunits, which are located between *trnF* and *trnL(UUR)* and separated by the *trnV* gene. The lengths and A + T contents of the *rrnS* and *rrnL* in the *Crossoptilon* genomes are within the range observed in other Phasianidae species. The rrnS contains three domains with 46 predicted stems, and the rrnL contains six domains with 59 stems (Additional files 4 and 5). The secondary structures of the rrnS in *C. crossoptilon* and *C. harmani* are identical; the rrnL secondary structures differ by only 2 bp in length. However, there are many differences in the rrnL secondary structures among the four *Crossoptilon* species, specifically in the loop near stem 44, which contains several substitutions and indels.

The complete mitogenome sequence contains 22 interspersed tRNA genes. All the tRNA sequences have the potential to fold into typical cloverleaf secondary structures except for *trnS(AGY)*, which lacks the DHU arm (Additional file 6). The secondary structures of *trnF*, *trnL(UUR)*, *trnQ*, *trnM*, *trnC*, *trnY*, *trnS(UCN)*, *trnH*, *trnL(CUN)*, *trnT*, and *trnP* are relatively conserved. However, the structure of *trnS(AGY)* is different in *C. auritum* relative to the other three *Crossoptilon* species (Additional file 6); the structure in *C. harmani*, *C. mantchuricum* and *C. crossoptilon* contain two additional bases (G and A) in the amino acid acceptor arm. The most frequent mismatch is G-U; other mismatches are also present, including U-U in *trnM* and *trnG*, C-C in *trnD* and *trnL(UUR)*, C-U in *trnF* and *trnI*, and A-C in *trnS(AGY)*. The *C. crossoptilon* and *C. harmani* tRNA structures are almost identical, with the exception of 1 bp. The A + T content of the tRNA genes is 57.6% in *C. harmani* and *C. crossoptilon*, 57.4% in *C. mantchuricum* and *C. auritum*.

### Control region

The nucleotide composition of the control region in the *Crossoptilon* species has a bias against G, which is common in the mitogenome sense strand of vertebrates [42]. The control region of *Crossoptilon* is located in the conserved position between *trnE* and *trnF* and the length (1146 nucleotides) is conserved in all four species. The control region contains three domains: the ETAS Domain I (nt 1–312), Central Conserved Domain II (nt 313–780) and CSB Domain III (nt 781–1146). The A + T content of the three domains is similar in all the *Crossoptilon* species; Domains I, II, and III have higher contents of C and A, T, and A, respectively, relative to other bases. Domain III contains a higher percentage of A than Domain II, and Domain II has the highest G content among the three domains. The distribution of variable sites and conserved sites suggests that Domain II has relatively more conserved sites and Domain I has more variable sites compared to other domains.

The ETAS Domain I can be divided into two parts: part A, from nt 1–163 and part B, from nt 164–312. There are two conserved blocks in part A, ETAS1 (nt 64–126) and ETAS2 (nt 124–163), which are similar to motifs previously identified in other avian and mammalian species. The first block is perfectly conserved among the *Crossoptilon* species and has sequence similarity to the “goose hairpin” described in some phasianids and *Anas* species [43-46] (Additional file 7). The secondary structure of this hairpin is determined by a stem of seven complementary Cs/Gs and a loop containing a TCCC motif also present in mammalian control region [47], which was associated experimentally with termination of H strands [48]. There are two copies of TCCC located at nt 22–25 and 183–186. The second block, which is perfectly conserved among the *Crossoptilon* species, has sequence similarity to the mammalian TASs, including the highly conserved motif GTGCAT, which is present in all sequenced Phasianids and Anseriforms. The motif GYRCAT (Y = C/T; R = A/G) is widespread in Domain I of some mammalian control region [47], and it has been duplicated in the R1 repeats of many species, including the opossum [49] and several rodents [50,51]. Its functional importance is suggested by both comparative [47] and experimental [48] data. Single-stranded Domain I can form potential secondary cloverleaf structures. The first 190 nucleotides of Domain I of the *Crossoptilon* species can form stable secondary structures (Additional file 7). The configurations of the cloverleaves are similar in *C. harmani* and *C. crossoptilon* and vary slightly in *C. mantchuricum* and *C. auritum*; the stem-loop structures of the cloverleaves always include the “goose hairpin”. The control region of *C. harmani* and *C. crossoptilon* have similar structures with five stems, while *C. mantchuricum* and *C. auritum* have only four.

The Central Conserved Domain II of the control region of the *Crossoptilon* species includes 468 conserved nucleotides, from the hyper-variable site at position 313 at the end of the ETAS domain to the beginning of the putative O_H_ sequence block at position 781. Several conserved blocks of the central conserved domain are similar to the F (nt 350–377), E (nt 395–414), D (nt 455–479), and C (nt 504–529) boxes of vertebrates and other avian species [43,52], and a bird similarity box is also present.

The CSB Domain III is highly variable and has sequences similar to mammalian CSB1. A poly(C) sequence (nt 781–792), similar to the O_H_ of mammals, maps just a few nucleotides downstream from the putative CSB1 (nt 803–828). However, it is difficult to identify sequences corresponding to mammalian CSB2 and CSB3. The secondary structures of CSB1 and the putative goose hairpin in *Crossoptilon* are consistent with those of *G. gallus* (Additional file 7). The bidirectional LSP/HSP promoters (nt 982–1003) [53] are almost perfectly conserved among the *Crossoptilon* species. A stable hairpin (nt 1004–1017) that is rich in poly (T) and poly (A) strings is located immediately upstream of the promoters.

### Phylogeny and divergence time of the *Crossoptilon* species

**Figure 1:**
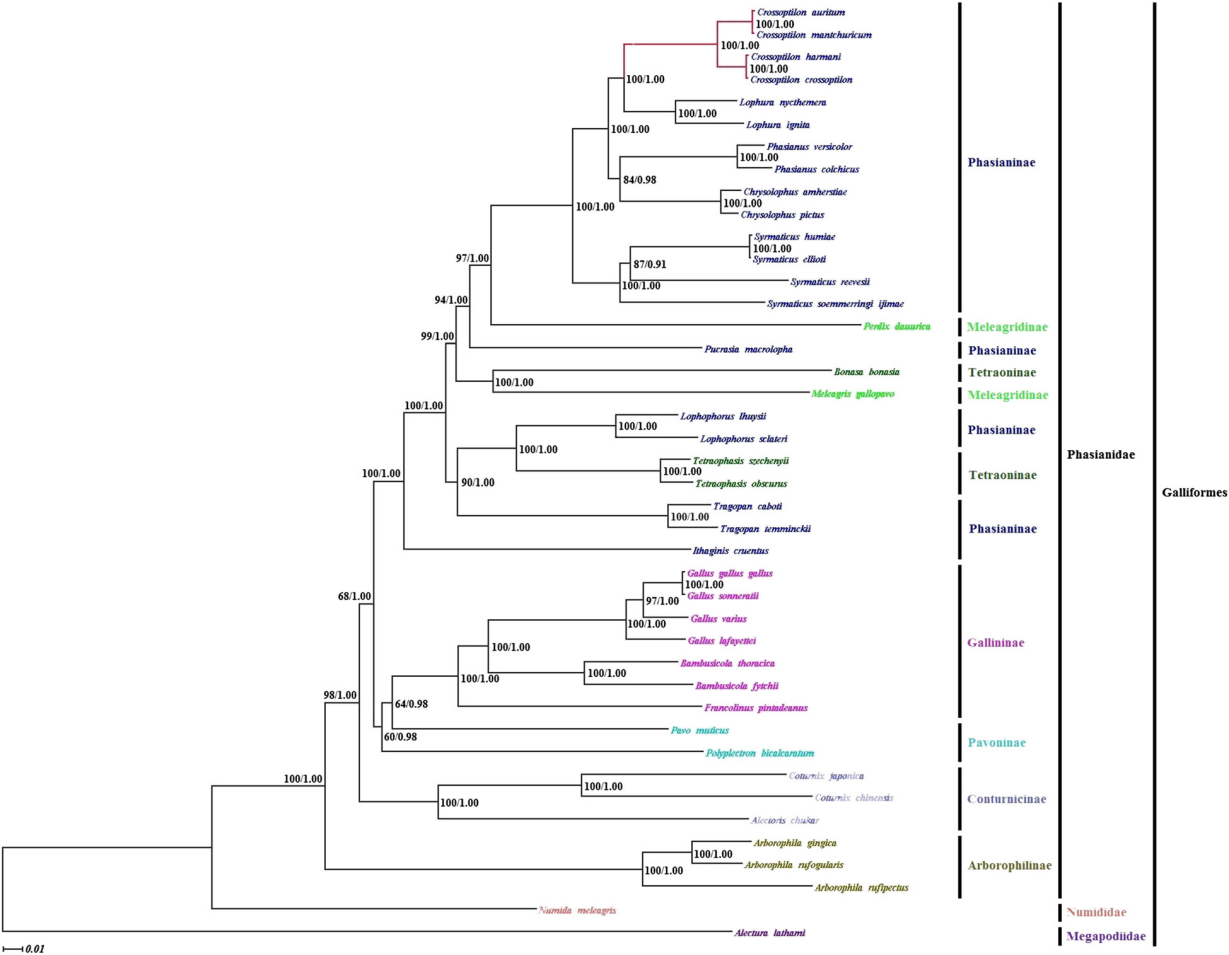
**The phylogenetic trees based on the PCG dataset using ML and BI methods.** Branch lengths and topologies were obtained using Bayesian inference analyses. Among the *Crossoptilon*, *C. auritum* with *C. mantchuricum* and *C. harmani* with *C. crossoptilon* form two pairs of sister groups.

The final combined PCG dataset has 11376 characters after alignment. For this dataset, parsimony analysis shows a length of 27412 steps, with CI =0.313, RI = 0.499. The topologies between ML and BI trees of PCG dataset were consistent (Figure 1). For the mitogenome dataset, there was no difference in topology between the ML and BI trees of Galliformes (Additional file 8); however, both trees differed from the MP tree (Additional file 8). According to the phylogenetic results, the monophyly of *Crossoptilon* was strongly supported in MP, ML and BI analyses. Within Phasianidae, the topology ((*Crossoptilon* + *Lophura*) + (*Phasianus* + *Chrysolophus*)) was formed in most trees. The sister-group relationship between *Crossoptilon* and *Lophura* was supported with bootstrap values 69 and 100 in MP and ML trees, and posterior probabilities 1.00 in BI tree of PCG dataset.

The genetic distances of the mitogenomes (Additional file 9) are identical (0.026) for *C. auritum* and *C. harmani*, *C. auritum* and *C. crossoptilon*, *C. harmani* and *C. mantchuricum*, and *C. mantchuricum* and *C. crossoptilon*. However, the genetic distances between *C. harmani* and *C. crossoptilon*, and *C. auritum* and *C. mantchuricum* are only 0.001 and 0.002, respectively. The genetic distances based on four single genes show that the genetic distances of *C. harmani*-*C. crossoptilon* and *C. auritum*-*C. mantchuricum* are much smaller. This value is similar to the intraspecific level and far less than the interspecific level (Additional file 9).

**Figure 2:**
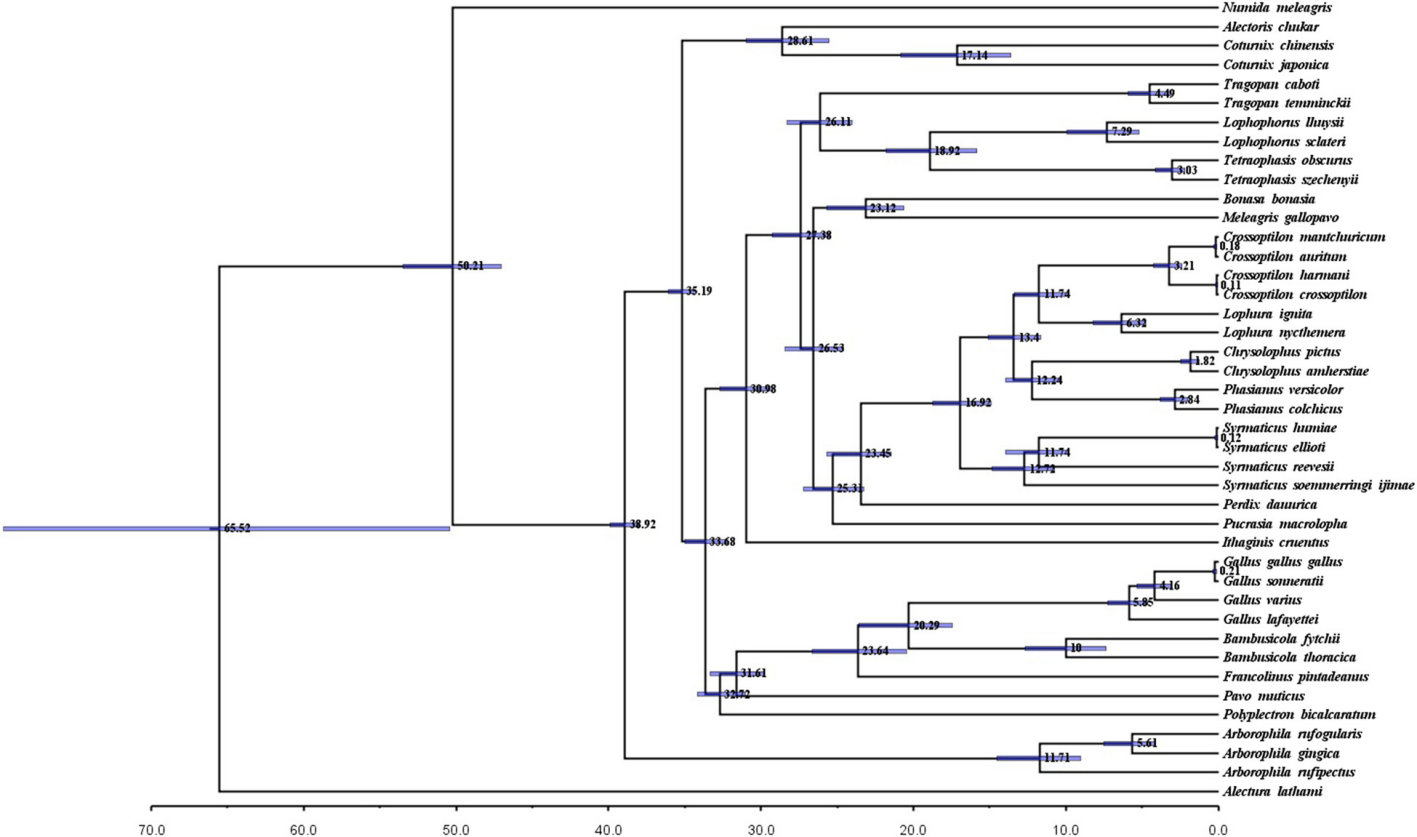
**The divergence time of** ***Crossoptilon*** **with 95% highest posterior-probability density.** Numbers above branches refer to divergence times. The *C. harmani*-*C. crossoptilon* and *C. mantchuricum*-*C. auritum* splits occurred at 3.21 Mya (95% HPD = 2.36-4.23 Mya). The divergence time for *C. harmani* and *C. crossoptilon* to be approximately 0.11 Mya (95% HPD = 0.05-0.18 Mya) and for *C. mantchuricum* and *C. auritum*, 0.18 Mya (95% HPD = 0.1-0.29 Mya).

According to the estimated timescale obtained from the phylogenetic tree containing consistent topology with PCG-ML/BI trees, the *C. harmani*-*C. crossoptilon* and *C. mantchuricum*-*C. auritum* splits occurred at 3.21 Mya (95% highest posterior-probability density (HPD) = 2.36-4.23 Mya) (Figure 2). The divergence time for *C. harmani* and *C. crossoptilon* is approximately 0.11 Mya (95% HPD = 0.05-0.18 Mya) and for *C. mantchuricum* and *C. auritum*, 0.18 Mya (95% HPD = 0.1-0.29 Mya) (Figure 2).

### Nonsynonymous and synonymous substitution

The analysis of variable sites in each protein-coding gene showed that the group *C.har*-*C.cro* and *C.man*-*C.aur* contained less variable sites, while other four groups included more (Additional file 10). The percentages of variable sites were higher in *nad3* and *nad6* genes among the groups *C.har*-*C.man*, *C.man*-*C.cro*, *C.har*-*C.aur* and *C.cro*-*C.aur*, while the percentages were lower in *atp8* and *atp6* genes. The Ka/Ks value of *atp8* in the five groups is far greater than 1 except for *C.har*-*C.cro*, and *atp6* and *cytb* in *C.har*-*C.cro* (Table 2), which show a strong positive selection. The Ka/Ks values of the other genes analysed in *Crossoptilon* species were less than 1, which shows a purifying selection (Table 2). Furthermore, the P-value (Fisher exact test) is much less than 0.001, except for *atp8* in *C.har*-*C.man*, *C.man*-*C.cro*, *C.har*-*C.aur* and *C.cro*-*C.aur* (0.558274 or 0.289204, respectively, which are obviously higher than 0.05) and *nad3* in *C.har*-*C.cro* (0.154), indicating that the difference is significant.

**Table 2:** **The Ka/Ks values among the** ***Crossoptilon*** **species**

**Gene**	***C.har*** **-** ***C.man***	***C.man*** **-** ***C.cro***	***C.har*** **-** ***C.cro***	***C.har*** **-** ***C.aur***	***C.man*** **-** ***C.aur***	***C.cro*** **-** ***C.aur***
*nad1*	0.01866	0.01866	0.750571	0.020942	1.00E-06	2.09E-02
*nad2*	0.034826	0.034826	0.7244	0.032325	1.00E-06	3.23E-02
*cox1*	1.00E-06	1.00E-06	1.00E-06	1.00E-06	1.00E-06	1.00E-06
*cox2*	0.024999	0.024999	0.597845	0.021423	1.00E-06	2.14E-02
*atp8*	50	50	0.719187	50	50	50
*atp6*	0.121909	0.080132	50	0.133656	1.00E-06	8.76E-02
*cox3*	0.005999	0.007105	1.00E-06	0.005766	1.00E-06	6.79E-03
*nad3*	0.137238	0.135219	0.187567	0.121225	1.00E-06	1.22E-01
*nad4L*	1.00E-06	1.00E-06	6.97E-01	1.00E-06	6.93E-01	1.00E-06
*nad4*	0.034302	0.034302	0.723156	0.028535	0.082408	0.028535
*nad5*	0.022191	0.024554	1.00E-06	0.022117	1.00E-06	2.45E-02
*cytb*	0.017382	0.011092	50	0.017382	0.725975	0.011092
*nad6*	0.044412	0.044412	0.683241	0.044412	0.685339	0.044412

Note: *C. harmani*: *C.har*, *C. mantchuricum*: *C.man*, *C. crossoptilon*: *C.cro*, *C. auritum*: *C.aur*. Most Ka/Ks values of genes analysed in *Crossoptilon* species are less than 1.

Both *atp6* and *cytb* genes have one different base in the *C.har*-*C.cro* group, i.e., nt 245 in *atp6* gene (T base or Phe in *C. harmani*, while C base or Ser in *C. crossoptilon*) and nt 709 in *cytb* gene (C base or Leu in *C. harmani*, while T base or Phe in *C. crossoptilon*). The *atp8* gene in *C.man*-*C.aur* group also contains one different base (nt 124), G base in *C. mantchuricum*, while A base in *C. auritum*, and conrespondingly Val in *C. mantchuricum*, while Met in *C. auritum*. Further study showed that four amino acid sites in *atp8* (T at 11 position, I at 12 position, S at 39 position and V at 42 position) in the genus *Crossoptilon* were positively selected sites, based on Bayes Empirical Bayes (BEB) analysis. This four different amino acid sites (T, I, S and V) corresponded to nucleotide sequences of ACT, ATC, AGC and GTA in *C. harmani* and *C. crossoptilon*, ATT, ACC, AAC and GTA in *C. mantchuricum*, ATT, ACC, AAC and ATA in *C. auritum*. The further secondary structure analysis of the amino acid sequence of atp8 showed that T at 11 position and I at 12 position located at helix (IN- > OUT) domain in transmembrane (TM) protein, while S at 39 position and V at 42 position located at outside domain.

### PBS analyses

PBS analyses were performed to better understand the contributions of different parts of the mitogenomes to the genome phylogeny based on the mitogenome-ML tree. The relationships among the PBS value, length, singleton sites (S), parsimony informative sites (Pi), variable sites (V) and conserved sites (C) in different partitions are also studied. Ranking individual protein-coding genes by their respective contribution to the total PBS values shows that some genes, such as *nad5* and *nad4* provide a higher contribution compared to other markers, while *atp8* and *nad4L* contribute less. But analysis also shows that PBS values are roughly correlated with gene lengths. The variable sites are closely related to parsimony informative sites, which follow the same trend. The third codon has a high PBS value but with less conserved sites, while the tRNA gene cluster IQM has a lower PBS value with more conserved sites compared to other partitions.

The third codon has a high PBS value and therefore contributes most to the mitogenome-ML tree. The NADH genes have secondary high PBS, which might be the effect of the third codon in the relatively long sequence. To further understand the contribution of the 3rd codon, we reconstructed the phylogenetic tree using this single partition.

In the phylogenetic trees based on the third codon (Additional file 8), the structure of the ML tree is similar to the BI with the exception of *Phasianus*. The positions of *Pucrasia macrolopha*, *Bonasa bonasia*, *Meleagris gallopavo* and *Polyplectron bicalcaratum* in the 3rd-MP tree are different compared to the BI tree. The 3rd-BI tree is identical to the mitogenome-ML with relative high support except for the position of *Polyplectron bicalcaratum*.

## Discussion

### Phylogenetic relationship and divergence time of the *Crossoptilon* species

In this study, the phylogenetic trees based on different datasets support the monophyly of *Crossoptilon* and the close genetic relationships of *C. auritum* and *C. mantchuricum* [4,12,54,55], *C. harmani* and *C. crossoptilon* [1,4,8,14,55]. Our analysis of the gene sequences of *Crossoptilon* based on different datasets confirms that *Crossoptilon* is the sister group to *Lophura* [55,56], indicating their closer relationship.

The genetic distance is rather small among some *Crossoptilon* species, for instance, between *C. harmani* and *C. crossoptilon*, *C. auritum* and *C. mantchuricum*. Morphological, ecological and behavioural studies have revealed the high similarity between *C. harmani* and *C. crossoptilon*, but with different plumage coloration [1]. Their distributions are overlapping, and *C. harmani* routinely hybridises with *C. c. drouynii* [1], which indicates their reproductive compatibility. Furthermore, the distribution of *C. harmani* is limited between the Himalayan and Nyenchen Tanglha ranges, which provided the geographical isolation required for the formation of a subspecies or species. Therefore, the taxonomic status and its relationship to *C. crossoptilon* are much questionable, which need to be re-evaluated by multiple markers and population genetics in the further studies.

Based on their significant differences in many aspects such as morphology and behaviour, *C. auritum* and *C. mantchuricum* are commonly considered to be independent species. However, the genetic distance of 0.002 indicates that they have a much closer genetic relationship. Consistent with this observation, Tsam et al. (2003) [12] observed that the interspecific differentiation between *C. mantchuricum* and *C. auritum* (0.18%) is less than the degree of differentiation among the *C. crossoptilon* subspecies.

Some Phasianidae species were found in the Pliocene epoch, and several modern taxa had already appeared in the Quaternary Pleistocene. The earliest fossils of *Crossoptilon* were found in the Cenozoic Zhoukoudian strata of Beijing, and in the Pleistocene strata at Yanjinggou in Wanxian of Sichuan Prov. [57,58]. The evolution of *Crossoptilon* mainly occurred corresponding to the Tibetan Plateau intensive uplift during the Tertiary Pliocene and the alternation of the glacial and interglacial periods in the Quaternary, which created profound and complex changes in the geographical and ecological environments [59,60] and was thought to have greatly affected its topography and avian species diversification [61,62]. Based on our results, the spliting time between *C. harmani*-*C. crossoptilon* and *C. mantchuricum*-*C. auritum* is consistent with the uplift during the Tertiary Pliocene and the fossil record in the Sichuan Wanxian Yanjinggou Pleistocene strata, which is approximate to the divergence times estimated by Jiang et al. (2014) [63] (3.78 Mya, 95% HPD = 1.17-6.56 Mya). Our study shows that *C. mantchuricum* and *C. auritum* are relatively ancient groups, while *C. harmani* and *C. crossoptilon* diverged later; these observations are not consistent with the *Crossoptilon* evolution hypothesis proposed by Lu et al. (1998) [1].

Lu et al. (1998) [1] suggested that the ancestor of *C. crossoptilon* and *C. harmani* originated in Sichuan Prov., Yunnan Prov., and the Tibetan border area. They noted that the clade containing the *C. harmani* plesiomorphy migrated to the hinterland plateau and subsequently differentiated into the ancestor of *C. harmani* and *C. auritum*. The ancestor of *C. auritum* dispersed in the northern plateau, and differentiated into *C. auritum* and *C. mantchuricum*. Based on the divergence time and distribution characteristics of *Crossoptilon* species, our results support the ancestor of *Crossoptilon* was initially distributed in Sichuan Prov., Yunnan Prov., and the Tibetan border area. The *Crossoptilon* first diverged into the ancestor of *C. harmani*-*C. crossoptilon* and the ancestor of *C. auritum*-*C. mantchuricum*. Considering the geographical distribution of *Crossoptilon* [1,64-66], we propose that the ancestor of *C. harmani*-*C. crossoptilon* might migrated to platform of the plateau, and then splitted into two lineages during the last uplift of the Tibetan Plateau [67], one adapted the high altitude environment to form *C. harmani* in east Tibet, and the other evoluted into *C. crossoptilon,* while the ancestor of *C. auritum*-*C. mantchuricum* dispersed to the northern plateau and further differentiated nearly the same period with *C. harmani*-*C. crossoptilon*.

### Selective pressure on protein-coding genes in *Crossoptilon* species

The Ka/Ks values in most protein-coding genes are less than 1 (Ka is lower than Ks) with P-values less than 0.001, which indicates that these genes in *Crossoptilon* are under purifying selection. The Ka/Ks values of each gene among *C.har*-*C.man*, *C.man*-*C.cro*, *C.har*-*C.aur* and *C.cro*-*C.aur* are similar. The Ka/Ks value of the *atp8* gene is 50 in five groups except for *C.har*-*C.cro*, which reveals the strong positive selection. However, only the P-value for *C.man*-*C.aur* is far less than 0.01 (P = 0), which indicates significant differences. In contrast, the Ka/Ks values of the *atp6* and *cytb* genes in *C.har*-*C.cro* are 50 with P-values less than 0.001, which also indicates strong positive selection. Previous studies have shown that *atp6* gene is highly variable in the mitogenomes of humans living in extremely cold areas [68-71], the *atp6* gene faces strong selection pressure with increasing altitude [72], and the variation of *atp6* gene in *Artemia tibetiana* is the result of adaptation to the cold and hypoxic environment of the plateau [73]. The variation in *atp6* may reflect the adaptation of *C. harmani* to the plateau environment; *cytb* might also play an important role in this process. The *atp8* gene in *C. mantchuricum* and *C. auritum* may also have experienced a strong selection in adaption to the plateau environment.

However, the further study showed that only four amino acid sites of atp8 in the *Crossoptilon* are positively selected sites, and accordingly this gene may have experienced strong selective pressure for plateau adaptation. The corresponding TM-helix (IN- > OUT) or outside position might be important for the *Crossoptilon* adaptation. After discarding groups (*C.har*-*C.cro* and *C.man*-*C.aur*) and genes (*cox1*, *atp8* and *nad4l*) with obviously different Ka/Ks values to others, the average value (0.0431) is similar with the strongly locomotive group (0.04) in the study of Shen et al. (2009) [74], which indicates the *Crossoptilon* accumulates fewer nonsynonymous mutations and is corresponding to their strong running ability.

## Conclusions

In summary, the mitogenomes of the *Crossoptilon* species contain the same gene arrangements and similar compositions including base contents and secondary structures. According to the phylogenetic trees, *C. auritum* with *C. mantchuricum*, and *C. harmani* with *C. crossoptilon* form two pairs of sister groups. *Crossoptilon* have a closer relationship with *Lophura*. Based on the genetic distances, *C. harmani* is more closely related with *C. crossoptilon,* and is the most recent diverged descendent of *Crossoptilon* as a result of the plateau adaptation. According to the molecular dating results, the divergence time between *C. harmani*-*C. crossoptilon* and *C. mantchuricum*-*C. auritum* is consistent with the uplift of the Tibetan plateau and the subsequently climate change during the Tertiary Pliocene. The Ka/Ks analysis showed that *atp8* gene in the *Crossoptilon* species may have experienced strong selection for the plateau environment adaptation.

## Additional files

Additional file 1:
**PCR primers used in this study.** The mitogenome of *C. harmani* was amplified by seven parts, that is L1263x-H2891x, L2260x-H6681x, L5758x-H7122x, L6615x-H10884x, L10635x-H13563x, L13040x-H16064x and L15725x-H1530x. The *C. mantchuricum* mitogenome was amplified by 12 fragments, that is L1263x-H2891x, L2260x-H4644x, L3803x-H6681x, L5758x-H7122x, L6615x-H8121x, L7525x-H8628x, L8386x-H10884x, L10635x-H12344x, L11458x-H13563x, L13040x-H15646b, L14080x-H16064x and L15413b-H1530x. The *C. crossoptilon* mitogenome was amplified by 13 fragments, that is L1263x-H2891x, L2260x-H4644x, L3803x-H6681x, L5758x-H7122x, L6615x-H8121x, L7525x-H8628x, L8386x-H10884x, L10635x-H12344x, L11458x-H13563x, L12156b-H14127b, L13525b-H15049x, L14080x-H16064x and L15725x-H1530x. The gaps were completed by other adjacent primers. Additional file 2:
**Species studied in the phylogenetic analysis.**
Additional file 3:
**The single genes (** ***nad2*** **, CR,** ***cytb*** **and** ***rrnS*** **) used to calculate genetic distances among** ***Crossoptilon*** **species.**
Additional file 4:
**The predicted secondary structure of rrnS in** ***C. harmani*** **.** Nucleotide differences across all other three species are labled (*C. mantchuricum*: *C. man*, *C. crossoptilon*: *C. cro*, *C. auritum*: *C. aur*). Additional file 5:
**The predicted secondary structure of rrnL in** ***C. harmani*** **.** Nucleotide differences across all other three species are labled (*C. mantchuricum*: *C. man*, *C. crossoptilon*: *C. cro*, *C. auritum*: *C. aur*). Additional file 6:
**The secondary structure of all tRNAs in** ***C. harmani*** **.** Nucleotide differences across all other three species are labled (*C. mantchuricum*: *C. man*, *C. crossoptilon*: *C. cro*, *C. auritum*: *C. aur*). Additional file 7:
**The putative secondary structure of the control region in** ***Crossoptilon*** **.** Note: A: nt 1–190 in *C. crossoptilon* and *C. harmani*; B: nt 1–190 in *C. auritum* and *C. mantchuricum*. Additional file 8:
**The MP, ML and BI trees of different datasets. Less than 50% bootstrap values were omitted.** Notes: (1) results based on PCG dataset; (2) results based on mitogenome dataset; (3) results based on the third codons dataset (3rd). (A) MP tree; (B) ML tree; (C) BI tree. Additional file 9:
**Interspecific and intraspecific genetic distances of the** ***Crossoptilon*** **species based on mitogenome dataset and single genes.** Note: *C. harmani*: *C.har*, *C. mantchuricum*: *C. man*, *C. crossoptilon*: *C. cro*, *C. auritum*: *C. aur.*
Additional file 10:
**The variable sites of protein-coding genes among six** ***Crossoptilon*** **groups.** Note: *C. harmani*: *C.har*, *C. mantchuricum*: *C. man*, *C. crossoptilon*: *C. cro*, *C. auritum*: *C. aur.*

## Abbreviations

*atp6* and *atp8*: ATP synthase subunits 6 and 8
*cytb*: Cytochrome b
*cox1-3*: Cytochrome c oxidase subunit 1 to 3
*nad1*-6: NADH dehydrogenase, subunit 1–6
*nad4L*: NADH dehydrogenase, subunit 4L
*rrnS* and *rrnL*: Small and large ribosomal RNA (rRNA) subunits
CR: Control region

## References

[1] Lu X, Zheng GM, Gu BY (1998). A preliminary investigation on taxonomy, distribution and evolutionary relationship of the eared pheasants, *Crossoptilon*. Acta Zool Sin.

[2] Cheng TH (1994). A complete checklist of species and subspecies of the Chinese birds.

[3] Lu TC (1991). The rare and endangered wild chicken in Chain.

[4] Cheng TH, Tan YK, Lu TC, Tang CG, Bao GJ, Li FL (1978). Fauna of China. Aves, Vol. IV. Galliformes.

[5] Johnsgard PA (1986). The Pheasants of the World.

[6] Shi XD, Zhang ZW, Liu LY (2001). Karyo types and G-banding patterns of three eared-pheasant (*Crossoptilon*) species. Acta Zool Sin.

[7] Gan YL, Lu TC, Liu RS, He FQ, Lu CL, Gan YL, Lu TC, Liu RS, He FQ, Lu CL (1992). Observation on scanning electron microscope of eggshell of *C. mantchuricumis*, *C. crossoptilon* and *C. auritum* endemic pheasants in China. Acta Zool Sin.

[8] Zheng GM, Zhang W, Zhao XR, Gao W (1991). A comparative research on the hind limb muscles of eared pheasants, *Crossoptilon*. The Study of Birds in China.

[9] Lu TC, Liu RS, He FQ, Lu CL, Li GY (1989). Ecology and systematic relationship of three species of the genus *Crossoptilon*. Sichuan J Zool.

[10] Liu RS, Guo YJ, Li FL, Hou LH (1985). Study on the relationship among three species of the genus *Crossoptilon* by electraofocusing technique. Acta Zool Sin.

[11] Ludlow F, Kinnear NB (1944). The birds of south-eastern Tibet. Ibis.

[12] Tsam CDM, Rao G, Ji JG, Suo LCR, Wan QH, Fang SG (2003). Taxonomic status of *Crossoptilon harmani* and a phylogenetic study of the genus *crossoptilon*. Acta Zool Sin.

[13] Rothschild L (1926). On the avifauna of Yunnan, with critical notes. Novit Zool.

[14] Delacour J (1977). The Pheasant of the World.

[15] Wu AP, Ding W, Zhang ZW, Zhan XJ (2005). Phylogenetic relationship of the avian genus *Crossoptilon*. Acta Zool Sin.

[16] Yang C, Lei FM, Huang Y (2010). Sequencing and Analysis of the Complete Mitochondrial Genome of *Pseudopodoces humilis* (Aves, Paridae). Zool Res.

[17] Sorenson MD (2003). Avian mtDNA primers.

[18] Staden R, Beal KF, Bonfield JK (2000). The Staden package, 1998. Methods Mol Biol.

[19] Lowe TM, Eddy SR (1997). tRNAscan-SE: a program for improved detection of transfer RNA genes in genomic sequence. Nucleic Acids Res.

[20] Cannone JJ, Subramanian S, Schnare MN, Collett JR, D'Souza LM, Du Y, Feng B, Lin N, Madabusi LV, Müller KM, Pande N, Shang Z, Yu N, Gutell RR (2002). The Comparative RNA Web (CRW) site: an online database of comparative sequence and structure information for ribosomal, intron, and other RNAs. BMC Bioinformatics.

[21] Burk A, Douzery EJP, Springer MS (2002). The secondary structure of mammalian mitochondrial 16S rRNA molecules: refinements based on a comparative phylogenetic approach. J Mamm Evol.

[22] Tamura K, Dudley J, Nei M, Kumar S (2007). MEGA4: molecular evolutionary genetics analysis (MEGA) software version 4.0. Mol Biol Evol.

[23] Thompson JD, Gibson TJ, Plewniak F, Jeanmougin F, Higgins DG (1997). The CLUSTAL_X windows interface: flexible strategies for multiple sequence alignment aided by quality analysis tools. Nucleic Acids Res.

[24] Swofford DL (2003). PAUP*. Phylogenetic Analysis Using Parsimony (*and Other Methods), Version 4.

[25] Stamatakis A (2006). RAxML-VI-HPC: maximum likelihood-based phylogenetic analyses with thousands of taxa and mixed models. Bioinform.

[26] Ronquist F, Huelsenbeck JP (2003). MrBayes 3: Bayesian phylogenetic inference under mixed models. Bioinformatics.

[27] Nylander JAA. MrModeltest v2. Program distributed by the author. Evolutionary Biology Centre, Uppsala University;2004.

[28] Sorenson MD, Franzosa EA (2007). TreeRot, version 3.

[29] Drummond AJ, Rambaut A (2007). BEAST: bayesian evolutionary analysis by sampling trees. BMC Evol Biol.

[30] Drummond AJ, Suchard MA, Xie D, Rambaut A (2012). Bayesian phylogenetics with BEAUti and the BEAST 1.7. Mol Biol Evol.

[31] Drummond AJ, Ho SYW, Phillips MJ, Rambaut A (2006). Relaxed phylogenetics and dating with confidence. PLoS Biol.

[32] Dyke GJ, Gulas BE, Crowe TM (2003). Suprageneric relationships of galliform birds (Aves, Galliformes): a cladistic analysis of morphological characters. Zool J Linnean Soc.

[33] Crowe TM, Bowie RCK, Bloomer P, Mandiwana TG, Hedderson TAJ, Randi E, Pereira SL, Wakeling J (2006). Phylogenetics, biogeography and classification of, and character evolution in, gamebirds (Aves: Galliformes): effects of character exclusion, data partitioning and missing data. Cladistics.

[34] Tordoff HB, Macdonald JR (1957). A new bird (family Cracidae) from the early Oligocene of South Dakota. Auk.

[35] Brodkorb P (1964). Catalogue of fossil birds, part 2 (Anseriformes through Galliformes). Bull Florida State Mus Biol Sci.

[36] Mourer-Chauviré C (1992). The Galliformes (Aves) of the Phosphorites du Quercy (France): systematics and biogeography. Natur Hist Mus Los Angeles County Sci Ser.

[37] Zhang Z, Li J, Zhao XQ, Wang J, Wong GK, Yu J (2006). KaKs_Calculator: calculating Ka and Ks through model selection and model averaging. Genomics Proteomics Bioinforma.

[38] Nei M, Kumar S (2000). Molecular Evolution and Phylogenetics.

[39] Yang Z (2006). Computational Molecular Evolution.

[40] Xu B, Yang Z (2013). PAMLX: a graphical user interface for PAML. Mol Biol Evol.

[41] Bernsel A, Viklund H, Hennerdal A, Elofsson A (2009). TOPCONS: consensus prediction of membrane protein topology. Nucleic Acids Res.

[42] Wolstenholme DR (1992). Animal mitochondrial DNA: structure and evolution. Int Rev Cytol.

[43] Quinn TW (1992). The genetic legacy of Mother Goose–phylogeographic patterns of lesser snow goose Chen caerulescens caerulescens maternal lineages. Mol Ecol.

[44] Fumihito A, Miyake T, Sumi S-I, Takada M, Ohno S, Kondo N (1994). One subspecies of the red junglefowl (*Gallus gallus gallus*) suffices as the matriarchic ancestor of all domestic breeds. Proc Natl Acad Sci U S A.

[45] Fumihito A, Miyake T, Takada M, Ohno S, Kondo N (1995). The genetic link between the Chinese bamboo partridge (*Bambusicola thoracica*) and the chicken and junglefowls of the genus *Gallus*. Proc Natl Acad Sci U S A.

[46] Ramirez V, Savoie P, Morais R (1993). Molecular characterization and evolution of a duck mitochondrial genome. J Mol Evol.

[47] Douzery E, Randi E (1997). The mitochondrial control region of Cervidae: evolutionary patterns and phylogenetic contents. Mol Biol Evol.

[48] Dufresne C, Mignotte F, Guéride M (1996). The presence of tandem repeats and the initiation of replication in rabbit mitochondrial DNA. Eur J Biochem.

[49] Gemmell NJ, Western PS, Watson JM, Marshall-Graves JA (1996). Evolution of the mammalian mitochondrial control region—comparisons of control region sequences between monotreme and therian mammals. Mol Biol Evol.

[50] Stewart DT, Baker AJ (1994). Patterns of sequence variation in the mitochondrial D-loop region of shrews. Mol Biol Evol.

[51] Fumagalli L, Taberlet P, Favre L, Hausser J (1996). Origin and evolution of homologous repeated sequences in the mitochondrial DNA control region of shrews. Mol Biol Evol.

[52] Sbisà E, Tanzariello F, Reyes A, Pesole G, Saccone C (1997). Mammalian mitochondrial D-loop region structural analysis: identification of new conserved sequences and their functional and evolutionary implications. Gene.

[53] L’Abbé DL, Duhaime JF, Lang BF, Morais R (1991). The transcription of DNA in chicken mitochondria initiates from one major bidirectional promoter. J Biol Chem.

[54] Tang CZ (1998). The analysis of system classification and geographical distribution of *Crossoptilon*. Acta Zool Sin.

[55] Wang N, Kimball RT, Braun EL, Liang B, Zhang ZW (2013). Assessing phylogenetic relationships among Galliformes: a multigene phylogeny with expanded taxon sampling in Phasianidae. PLoS One.

[56] Shen YY, Dai K, Cao X, Murphy RW, Shen XJ, Zhang YP (2014). The Updated Phylogenies of the Phasianidae Based on Combined Data of Nuclear and Mitochondrial DNA. PLoS One.

[57] Wetmore A (1934). Fossil birds from Mongolia and China. Am Aus Novit.

[58] Hou LH (1982). Avian fossils of pleistocene from Zhoukoudian, China. Vertebrata Pal Asiatica.

[59] Yang YC, Li BY, Yin ZS, Zhang QS, Wang FB, Jing K, Cheng ZM (1983). Geomorphology of Xizang (Tibet).

[60] Li BY, Wang FB, Chinese Academy of Sciences (1986). Basic characteristics of landforms in the northwest Yunnan and southwest Sichuan area. The Comprehensive Scientific Expedition to the Qinghai-Xizang Plateau. Studies in Qinghai-Xizang (Tibet) Plateau Special Issue of Hengduan Mountains Scientific Expedition (II).

[61] Li JJ, Shi YF, Li BY (1995). Uplift of the Qinghai–Xizang (Tibet) Plateau and Global Change.

[62] Lei FM, Qu YH, Song G (2014). Species diversification and phylogeographical patterns of birds in response to the uplift of the Qinghai-Tibet Plateau and Quaternary glaciations. Curr Zool.

[63] Jiang LC, Wang GC, Peng R, Peng QK, Zou FD (2014). Phylogenetic and molecular dating analysis of Taiwan Blue Pheasant (*Lophura swinhoii*). Gene.

[64] Wang FL, Chen JM, Lai RX (1985). Studies on the ancient and modern geographical distribution of Brown-eared Pheasants. J Shanxi Univ.

[65] Zhang CA, Ding CQ (2008). The distribution pattern of the Galliformes in China. Acta Zool Sin.

[66] Wu MX, Wu JG, Kuang MS, Heng T (2011). Relationship between Geographic Distribution of Endemic Birds and Climatic Factors in China. Res Env Sci.

[67] Li JJ, Fang XM (1999). Uplift of the Tibetan Plateau and environmental changes. Chinese Sci Bull.

[68] Elson JL, Turnbull DM, Howell N (2004). Comparative genomics and the evolution of human mitochondrial DNA: assessing the effects of selection. Am J Hum Genet.

[69] Mishmar D, Ruiz-Pesini E, Golik P, Macaulay V, Clark AG, Hosseini S, Brandon M, Easley K, Chen E, Brown MD, Sukernik RI, Olckers A, Wallace DC (2003). Natural selection shaped regional mtDNA variation in humans. Proc Natl Acad Sci U S A.

[70] Coskun PE, Ruiz-Pesini E, Wallace DC (2003). Control region mtDNA variants: longevity, climatic adaptation, and a forensic conundrum. Proc Natl Acad Sci U S A.

[71] Bhopal RS, Rafnsson SB (2009). Could mitochondrial efficiency explain the susceptibility to adiposity, metabolic syndrome, diabetes and cardiovascular diseases in South Asian populations?. Int J Epidemiol.

[72] Gu ML, Wang YJ, Shi L, Zhang YB, Chu JY (2009). Comparison on mitochondrial atp6, atp8 and cytb genes between Chinese Tibetans in three different zones: detecting the signature of natural selection on mitochondrial genome. Hereditas (Beijing).

[73] Zhang HX, Luo QB, Sun J, Liu F, Wu G, Yu J, Wang WW (2013). Mitochondrial genome sequences of *Artemia tibetiana* and *A. urmiana*: assessing molecular changes for high plateau adaptation. Sci China Life Sci.

[74] Shen YY, Shi P, Sun YB, Zhang YP (2009). Relaxation of selective constraints on avian mitochondrial DNA following the degeneration of flight ability. Genome Res.

