# Supplementary material for: Comparative mitochondrial genomics and phylogenetic relationships of the Crossoptilon species (Phasianidae, Galliformes)
Source: BMC Genomics. 2015 Feb 5;16(1):42. doi: 10.1186/s12864-015-1234-9 (PMC4326528; doi:10.1186/s12864-015-1234-9)
Supplement: Additional file 1: — PCR primers used in this study. The mitogenome of C. harmani was amplified by seven parts, that is L1263x-H2891x, L2260x-H6681x, L5758x-H7122x, L6615x-H10884x, L10635x-H13563x, L13040x-H16064x and L15725x-H1530x. The C. mantchuricum mitogenome was amplified by 12 fragments, that is L1263x-H2891x, L2260x-H4644x, L3803x-H6681x, L5758x-H7122x, L6615x-H8121x, L7525x-H8628x, L8386x-H10884x, L10635x-H12344x, L11458x-H13563x, L13040x-H15646b, L14080x-H16064x and L15413b-H1530x. The C. crossoptilon mitogenome was amplified by 13 fragments, that is L1263x-H2891x, L2260x-H4644x, L3803x-H6681x, L5758x-H7122x, L6615x-H8121x, L7525x-H8628x, L8386x-H10884x, L10635x-H12344x, L11458x-H13563x, L12156b-H14127b, L13525b-H15049x, L14080x-H16064x and L15725x-H1530x. The gaps were completed by other adjacent primers. [file 12864_2015_1234_MOESM1_ESM.doc]

Additional file 1 - PCR primers used in this study.

| Name | Location | Sequences (5’-3’) | Size (bp) |
| --- | --- | --- | --- |
| L1263x | *trnF* | AAAGCATGGCACTGAA | 16 |
| L2260x | *rrnS* | CAAGGTAAGYGTACCGGAAGGTG | 23 |
| L3803x | *rrnL* | CTACGTGATCTGAGTTCAGACCG | 26 |
| L5758x | *nad2* | GGAGGHTGAATRGGHCTAAACCARAC | 26 |
| L6615x | *trnY* | CCTCTGTAAAAAGGACTACAGCC | 23 |
| L7525x | *cox1* | GTATGAGCCCACCAYATATTYAC | 23 |
| L8386x | *cox2* | GCCTCATCCCCCATCATAGAAGA | 23 |
| L10635x | *cox3* | CACCACTTCGGATTYGAAGCAGC | 23 |
| L11458x | *nad4L* | TCHACMCGAACACAYGGCTCHGA | 23 |
| L12156b | *nad4* | CCHAAAGCMCACGTAGAAGCMCC | 23 |
| L13040x | *trnL* | ATCCRTTGGTCTTAGGARCCA | 21 |
| L13525b | *nad5* | GMTGAGAAGGRGTAGGAATCATATC | 25 |
| L14080x | *nad5* | TCAACYCATGCMTTCTTYAARGC | 23 |
| L15413b | *cytb* | GGWGGATTYTCAGTAGACAACCC | 23 |
| L15725x | *cytb* | AAACCAGAATGRTAYTTYCTATTYGC | 26 |
| H1530x | *rrnS* | GGTGGCTGGCACAAGATTTACC | 22 |
| H2891x | *rrnL* | TGRTGGCTGCTTKAAGGCCCAC | 22 |
| H4644x | *nad1* | TCRAATGGGGCRCGGTTRGTTTC | 26 |
| H6681x | *cox1* | GGTAAAGAGTGCCAATRTCTTTRTG | 25 |
| H7122x | *cox1* | ATGGTRGTGATRAAGTTGATDGCYCC | 26 |
| H8121x | *cox1* | GGGCAGCCRTGRATTCATTC | 20 |
| H8628x | *cox2* | TCRTAGGTTCAGTATCATTGRTGDCC | 26 |
| H10884x | *nad3* | GGGTCAAATCCRCATTCRTAYGG | 23 |
| H12344x | *nad4* | CTATGTGGCTHACRGAYGAGTARGC | 25 |
| H13563x | *nad5* | TGDAGTGCRGCTGTRTTRGC | 20 |
| H14127b | *nad5* | CCTATTTTTCGRATGTCYTGTTC | 23 |
| H15049x | *cytb* | GTGTCTGCDGTGTARTGYATKGC | 23 |
| H15646b | *cytb* | GGYGTGAARTTTTCTGGRTCTCC | 23 |
| H16064x | *trnT* | CTTCAGTTTTTGGTTTACAAGACC | 24 |

The mitogenome of *C. harmani* was amplified by seven parts, that is L1263x-H2891x, L2260x-H6681x, L5758x-H7122x, L6615x-H10884x, L10635x-H13563x, L13040x-H16064x and L15725x-H1530x. The *C. mantchuricum* mitogenome was amplified by 12 fragments, that is L1263x-H2891x, L2260x-H4644x, L3803x-H6681x, L5758x-H7122x, L6615x-H8121x, L7525x-H8628x, L8386x-H10884x, L10635x-H12344x, L11458x-H13563x, L13040x-H15646b, L14080x-H16064x and L15413b-H1530x. The *C. crossoptilon* mitogenome was amplified by 13 fragments, that is L1263x-H2891x, L2260x-H4644x, L3803x-H6681x, L5758x-H7122x, L6615x-H8121x, L7525x-H8628x, L8386x-H10884x, L10635x-H12344x, L11458x-H13563x, L12156b-H14127b, L13525b-H15049x, L14080x-H16064x and L15725x-H1530x. The gaps were completed by other adjacent primers.
