# Supplementary material for: Comparative mitochondrial genomics and phylogenetic relationships of the Crossoptilon species (Phasianidae, Galliformes)
Source: BMC Genomics. 2015 Feb 5;16(1):42. doi: 10.1186/s12864-015-1234-9 (PMC4326528; doi:10.1186/s12864-015-1234-9)
Supplement: Additional file 2: — Species studied in the phylogenetic analysis. [file 12864_2015_1234_MOESM2_ESM.doc]

Additional file 2 - Species studied in the phylogenetic analysis.

| Order | Family | Subfamily | Scientific name | Accession No. | Length(bp) |
| --- | --- | --- | --- | --- | --- |
|  |  |  | *Chrysolophus amherstiae* | FJ752434 | 16677 |
|  |  |  | *Chrysolophus pictus* | NC_014576 | 16678 |
|  |  |  | *Crossoptilon auritum* | NC_015897 | 16687 |
|  |  |  | *Crossoptilon crossoptilon* | KP259808 | 16680 |
|  |  |  | *Crossoptilon harmani* | KP259806 | 16682 |
|  |  |  | *Crossoptilon mantchuricum* | KP259807 | 16690 |
|  |  |  | *Ithaginis cruentus* | NC_018033 | 16683 |
|  |  |  | *Lophophorus lhuysii* | NC_013979 | 16712 |
|  |  |  | *Lophophorus sclateri* | FJ752432 | 16707 |
|  |  |  | *Lophura ignita* | NC_010781 | 16688 |
|  |  | Phasianinae | *Lophura nycthemera* | NC_012895 | 16680 |
|  |  |  | *Phasianus colchicus* | NC_015526 | 16692 |
|  |  |  | *Phasianus versicolor* | NC_010778 | 16690 |
|  |  |  | *Pucrasia macrolopha* | FJ752429 | 16696 |
|  |  |  | *Syrmaticus ellioti* | NC_010771 | 16688 |
|  |  |  | *Syrmaticus humiae* | NC_010774 | 16686 |
|  |  |  | *Syrmaticus reevesii* | NC_010770 | 16678 |
|  | Phasianidae |  | *Syrmaticus soemmerringi ijimae* | NC_010767 | 16690 |
|  |  |  | *Tragopan caboti* | NC_013619 | 16727 |
|  |  |  | *Tragopan temminckii* | FJ752427 | 16728 |
|  |  | Meleagridinae | *Meleagris gallopavo* | EF153719 | 16717 |
| Galliformes |  |  | *Perdix dauurica* | FJ752431 | 16695 |
|  |  | Tetraoninae | *Bonasa bonasia* | FJ752435 | 16673 |
|  |  |  | *Tetraophasis obscures* | NC_018034 | 16707 |
|  |  |  | *Tetraophasis szechenyii* | FJ752428 | 16709 |
|  |  | Gallininae | *Gallus gallus gallus* | NC_007236 | 16785 |
|  |  |  | *Gallus lafayetii* | NC_007239 | 16841 |
|  |  |  | *Gallus sonneratii* | AP006746 | 16783 |
|  |  |  | *Gallus varius* | NC_007238 | 16783 |
|  |  |  | *Bambusicola fytchii* | FJ752423 | 16726 |
|  |  |  | *Bambusicola thoracica* | NC_011816 | 16726 |
|  |  |  | Francolinus pintadeanus | NC_011817 | 16694 |
|  |  | Conturnicinae | *Alectoris chukar* | FJ752426 | 16686 |
|  |  |  | *Coturnix chinensis* | NC_004575 | 16687 |
|  |  |  | *Coturnix japonica* | NC_003408 | 16697 |
|  |  | Pavoninae | *Pavo muticus* | NC_012897 | 16698 |
|  |  |  | *Polyplectron bicalcaratum* | NC_012900 | 16702 |
|  |  | Arborophilinae | *Arborophila gingica* | FJ752425 | 16728 |
|  |  |  | Arborophila rufipectus | NC_012453 | 16728 |
|  |  |  | *Arborophila rufogularis* | FJ752424 | 16726 |
|  | Numididae |  | *Numida meleagris* | NC_006382 | 16726 |
|  | Megapodiidae |  | *Alectura lathami* | NC_007227 | 16698 |
