# Supplementary material for: Comparative mitochondrial genomics and phylogenetic relationships of the Crossoptilon species (Phasianidae, Galliformes)
Source: BMC Genomics. 2015 Feb 5;16(1):42. doi: 10.1186/s12864-015-1234-9 (PMC4326528; doi:10.1186/s12864-015-1234-9)
Supplement: Additional file 3: — The single genes ( nad2 , CR, cytb and rrnS ) used to calculate genetic distances among Crossoptilon species. [file 12864_2015_1234_MOESM3_ESM.doc]

Additional file 3 - The single genes (*nad2*, CR, *cytb* and *rrnS*) used to calculate genetic distances among *Crossoptilon* species.

| *nad2* | CR | *cytb* | *rrnS* |
| --- | --- | --- | --- |
| EU845772 *Crossoptilon auritum* | EU680828 *Crossoptilon auritum* | EU839479 *Crossoptilon auritum* | KC778966 *Crossoptilon auritum* |
| EU845771 *Crossoptilon auritum* | EU680827 *Crossoptilon auritum* | EU839478 *Crossoptilon auritum* | NC_015897 *Crossoptilon auritum* |
| KC778815 *Crossoptilon auritum* | JN684028 *Crossoptilon auritum* | AF534552 *Crossoptilon auritum* | KC749453 *Crossoptilon crossoptilon* |
| KC462594 *Crossoptilon auritum* | JN684027 *Crossoptilon auritum* | NC_015897 *Crossoptilon auritum* | NC_016679 *Crossoptilon crossoptilon* |
| KC462593 *Crossoptilon auritum* | JN684026 *Crossoptilon auritum* | AF028794 *Crossoptilon crossoptilon* | KP259808 *Crossoptilon crossoptilon* |
| KC462592 *Crossoptilon auritum* | AY343523 *Crossoptilon auritum* | GU214282 *Crossoptilon crossoptilon* | KC778967 *Crossoptilon harmani* |
| KC462591 *Crossoptilon auritum* | NC_015897 *Crossoptilon auritum* | NC_016679 *Crossoptilon crossoptilon* | KP259806 *Crossoptilon harmani* |
| KC462590 *Crossoptilon auritum* | JN684025 *Crossoptilon crossoptilon* | KP259808 *Crossoptilon crossoptilon* | KC778968 *Crossoptilon mantchuricum* |
| KC462589 *Crossoptilon auritum* | JN684024 *Crossoptilon crossoptilon* | AY343524 *Crossoptilon harmani* | KP259807 *Crossoptilon mantchuricum* |
| KC462588 *Crossoptilon auritum* | JN684023 *Crossoptilon crossoptilon* | GU214283 *Crossoptilon harmani* |  |
| KC462587 *Crossoptilon auritum* | AJ298921 *Crossoptilon crossoptilon* | KP259806 *Crossoptilon harmani* |  |
| KC462586 *Crossoptilon auritum* | AY343525 *Crossoptilon crossoptilon* | AF534553 *Crossoptilon mantchuricum* |  |
| KC462585 *Crossoptilon auritum* | NC_016679 *Crossoptilon crossoptilon* | KP259807 *Crossoptilon mantchuricum* |  |
| KC462584 *Crossoptilon auritum* | KP259808 *Crossoptilon crossoptilon* |  |  |
| NC_015897 *Crossoptilon auritum* | AY343521 *Crossoptilon harmani* |  |  |
| DQ307005 *Crossoptilon crossoptilon* | KP259806 *Crossoptilon harmani* |  |  |
| GU214315 *Crossoptilon crossoptilon* | EU747236 *Crossoptilon mantchuricum* |  |  |
| GU214314 *Crossoptilon crossoptilon* | EU747235 *Crossoptilon mantchuricum* |  |  |
| DQ768256 *Crossoptilon crossoptilon* | DQ987483 *Crossoptilon mantchuricum* |  |  |
| NC_016679 *Crossoptilon crossoptilon* | AY343522 *Crossoptilon mantchuricum* |  |  |
| KP259808 *Crossoptilon crossoptilon* | KP259807 *Crossoptilon mantchuricum* |  |  |
| KC778816 *Crossoptilon harmani* |  |  |  |
| KP259806 *Crossoptilon harmani* |  |  |  |
| KC778817 *Crossoptilon mantchuricum* |  |  |  |
| KP259807 *Crossoptilon mantchuricum* |  |  |  |
