# Supplementary material for: Comparative mitochondrial genomics and phylogenetic relationships of the Crossoptilon species (Phasianidae, Galliformes)
Source: BMC Genomics. 2015 Feb 5;16(1):42. doi: 10.1186/s12864-015-1234-9 (PMC4326528; doi:10.1186/s12864-015-1234-9)
Supplement: Additional file 4: — The predicted secondary structure of rrnS in C. harmani . Nucleotide differences across all other three species are labled (C. mantchuricum: C. man, C. crossoptilon: C. cro, C. auritum: C. aur). [file 12864_2015_1234_MOESM4_ESM.doc]

**
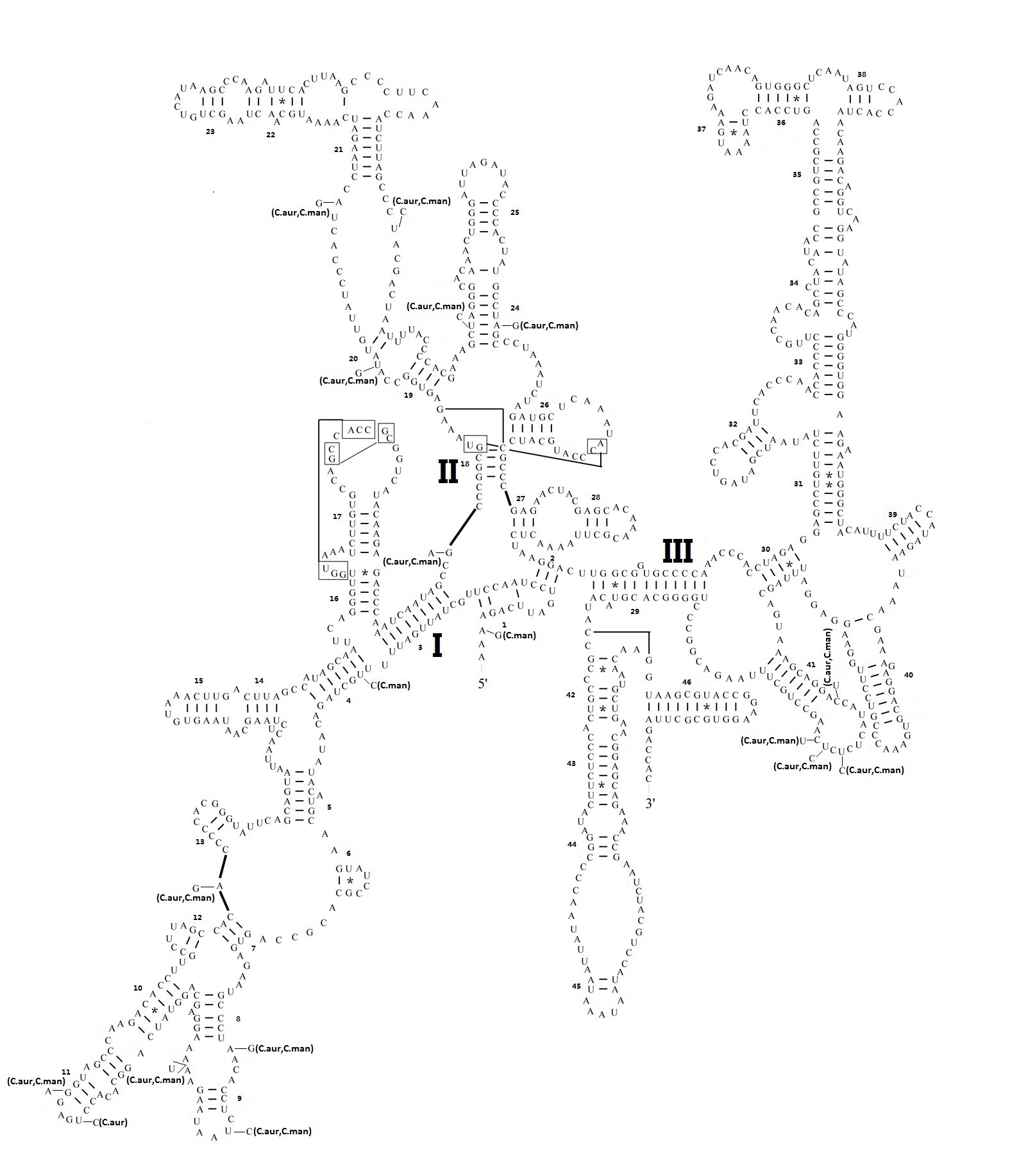
**

Additional file 4 - The predicted secondary structure of rrnS in *C. harmani*.

Nucleotide differences across all other three species are labled (*C. mantchuricum*: *C. man*, *C. crossoptilon*: *C. cro*, *C. auritum*: *C. aur*).
