# Supplementary material for: Comparative mitochondrial genomics and phylogenetic relationships of the Crossoptilon species (Phasianidae, Galliformes)
Source: BMC Genomics. 2015 Feb 5;16(1):42. doi: 10.1186/s12864-015-1234-9 (PMC4326528; doi:10.1186/s12864-015-1234-9)
Supplement: Additional file 7: — The putative secondary structure of the control region in Crossoptilon . Note: A: nt 1–190 in C. crossoptilon and C. harmani; B: nt 1–190 in C. auritum and C. mantchuricum. [file 12864_2015_1234_MOESM7_ESM.doc]

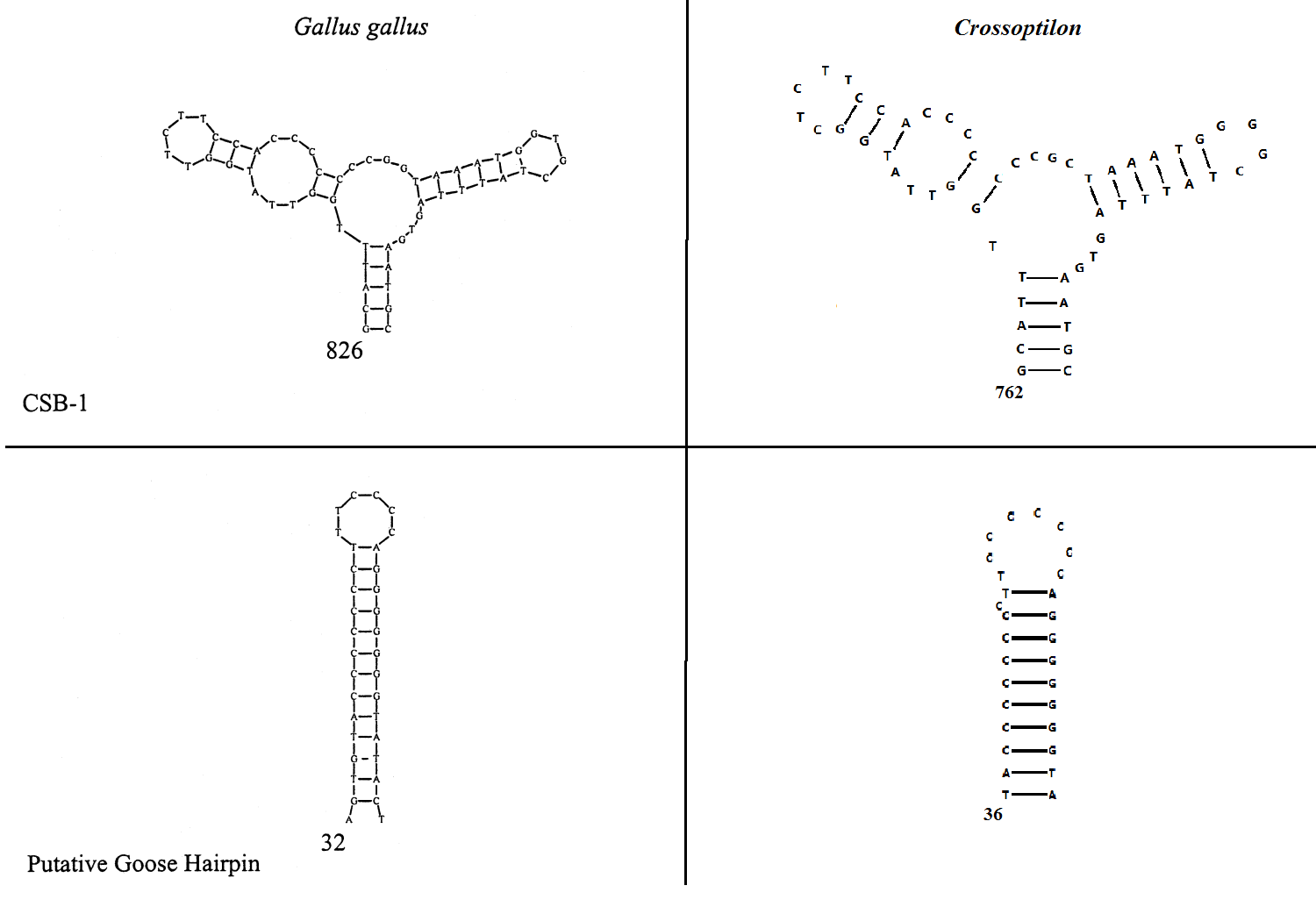


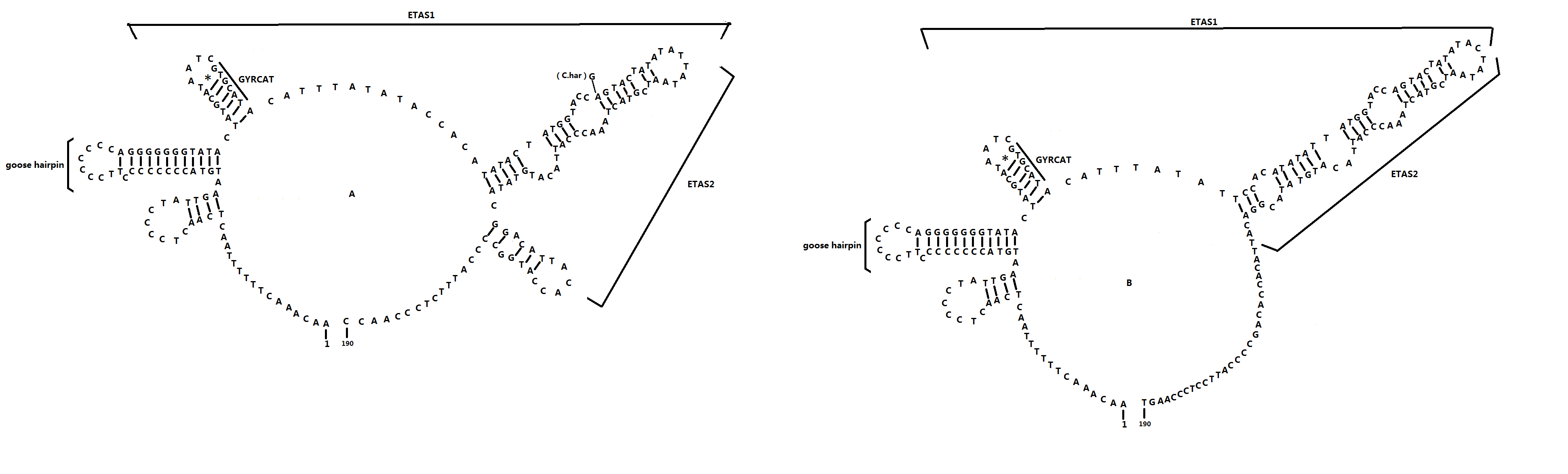


Additional file 7 - The putative secondary structure of the control region in *Crossoptilon*.

Note: A: nt 1-190 in *C. crossoptilon* and *C. harmani*; B: nt 1-190 in *C. auritum* and *C. mantchuricum*.
