# Supplementary material for: Comparative mitochondrial genomics and phylogenetic relationships of the Crossoptilon species (Phasianidae, Galliformes)
Source: BMC Genomics. 2015 Feb 5;16(1):42. doi: 10.1186/s12864-015-1234-9 (PMC4326528; doi:10.1186/s12864-015-1234-9)
Supplement: Additional file 8: — The MP, ML and BI trees of different datasets. Less than 50% bootstrap values were omitted. Notes: (1) results based on PCG dataset; (2) results based on mitogenome dataset; (3) results based on the third codons dataset (3rd). (A) MP tree; (B) ML tree; (C) BI tree. [file 12864_2015_1234_MOESM8_ESM.doc]

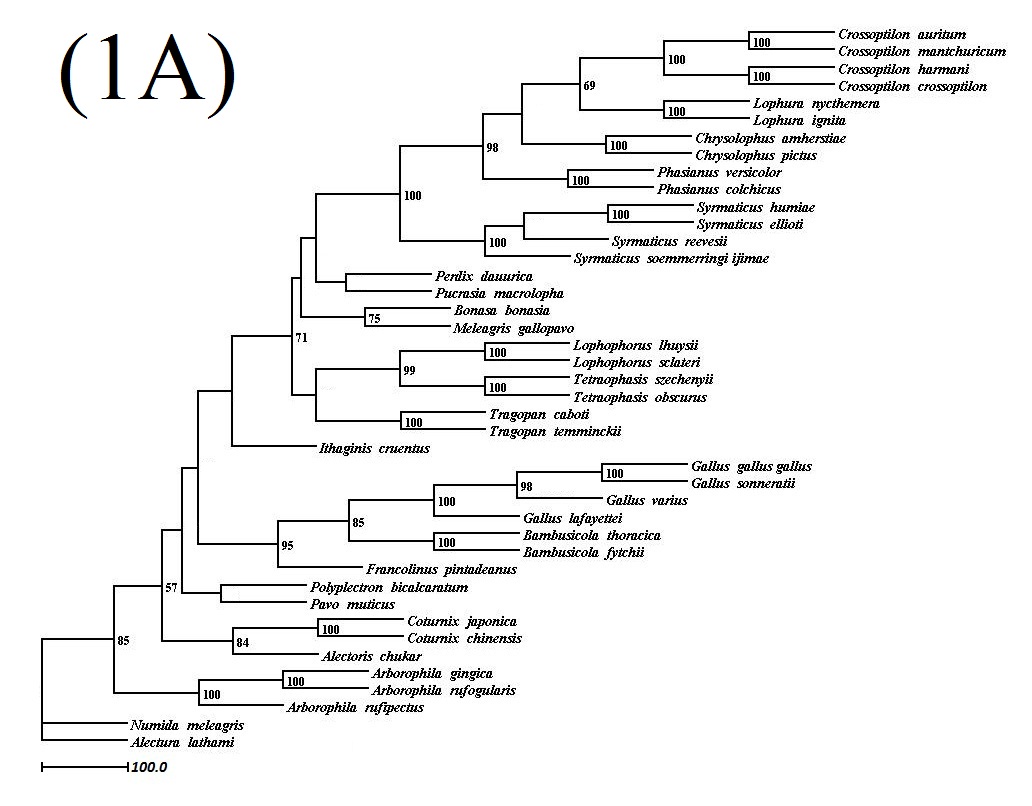


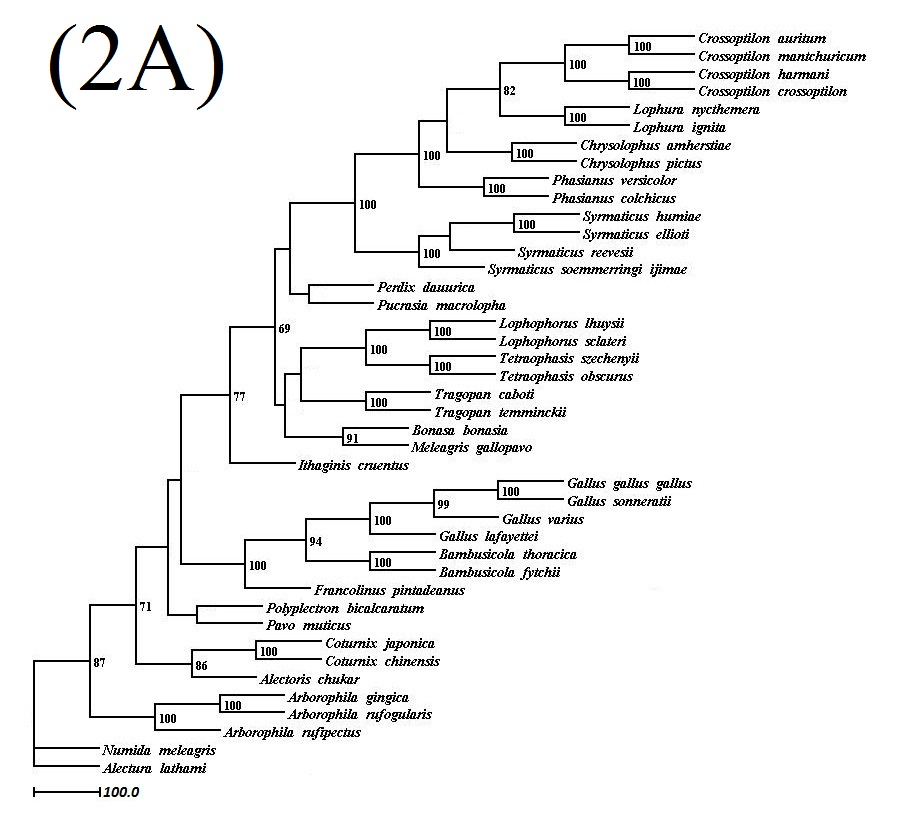


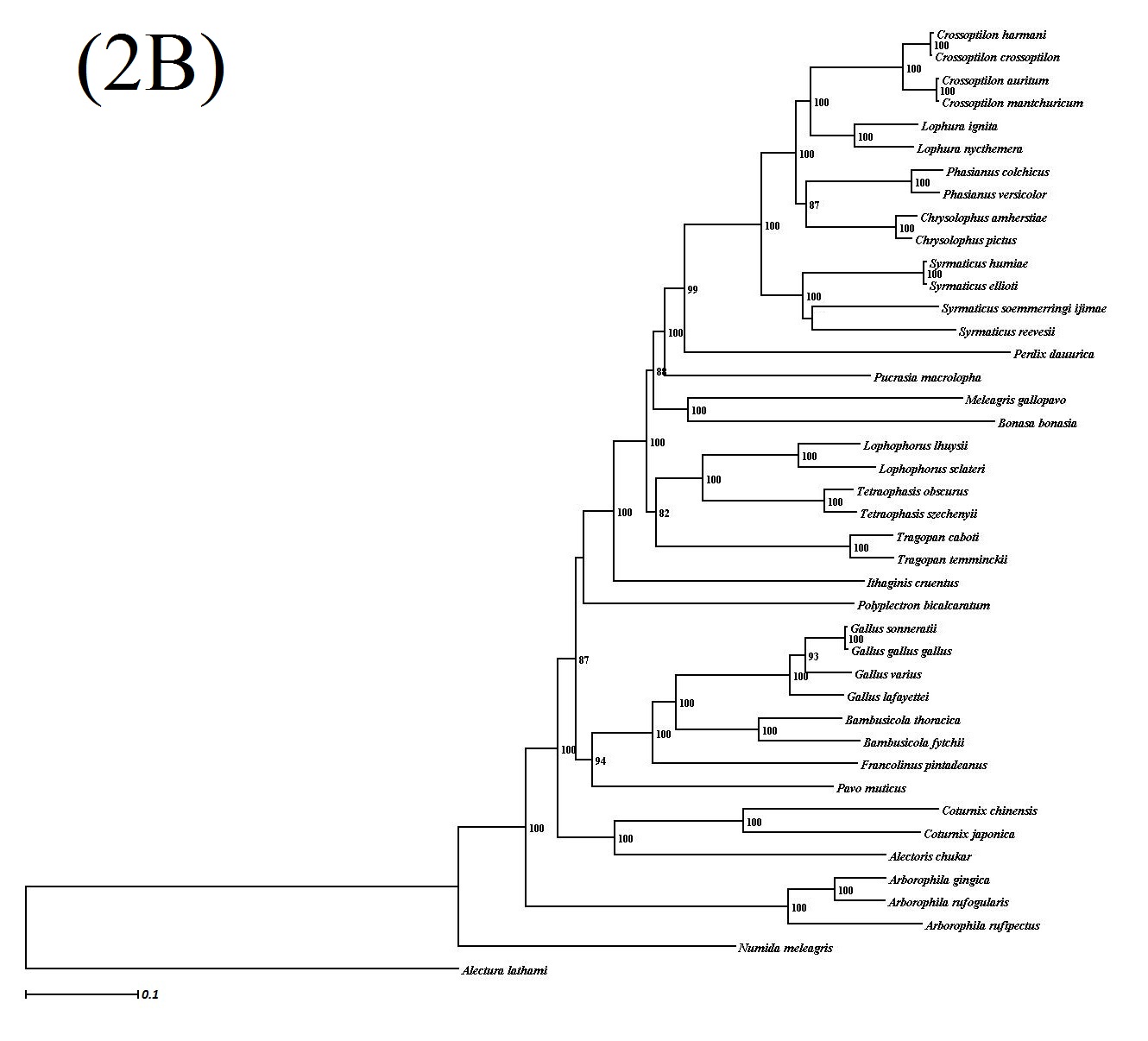


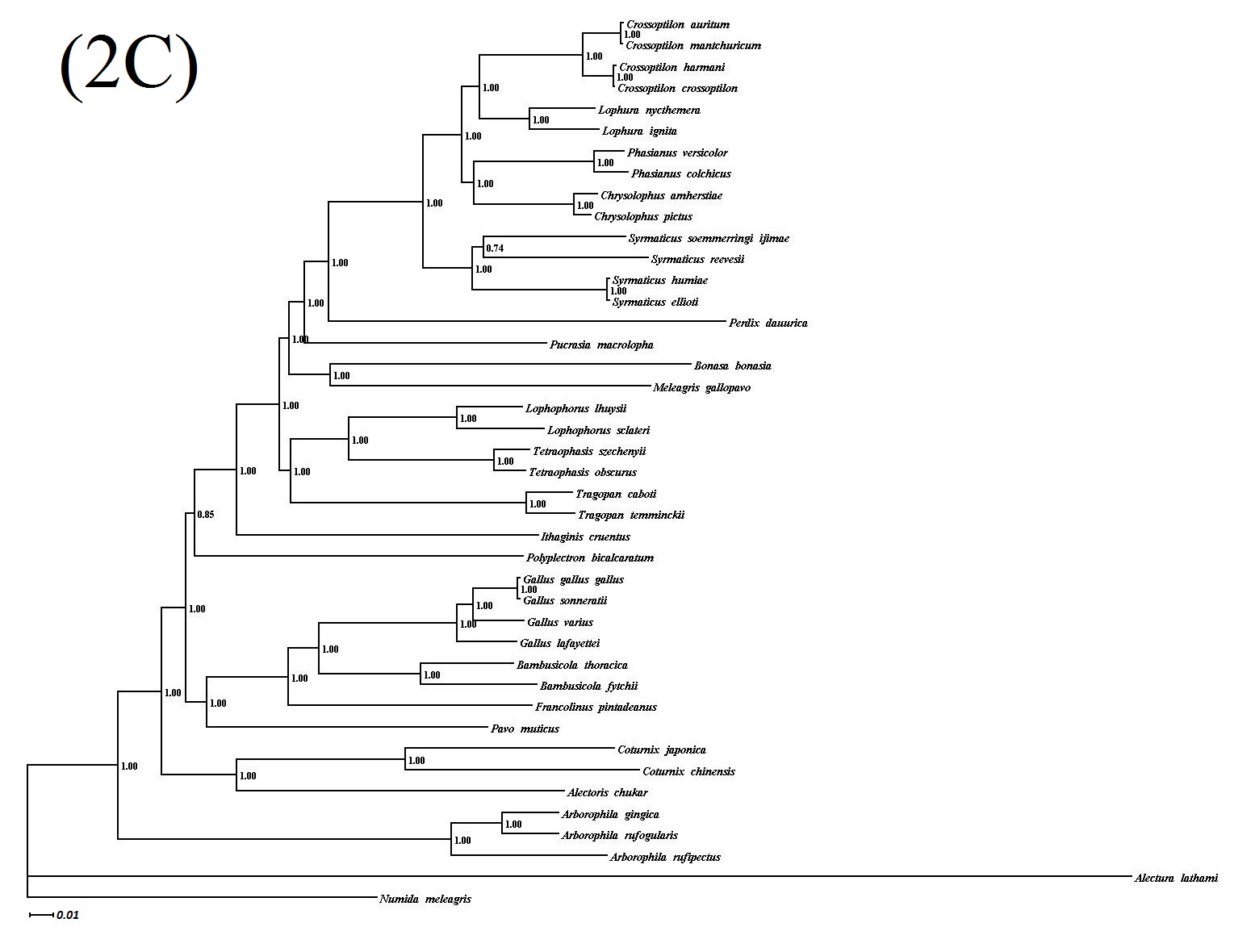


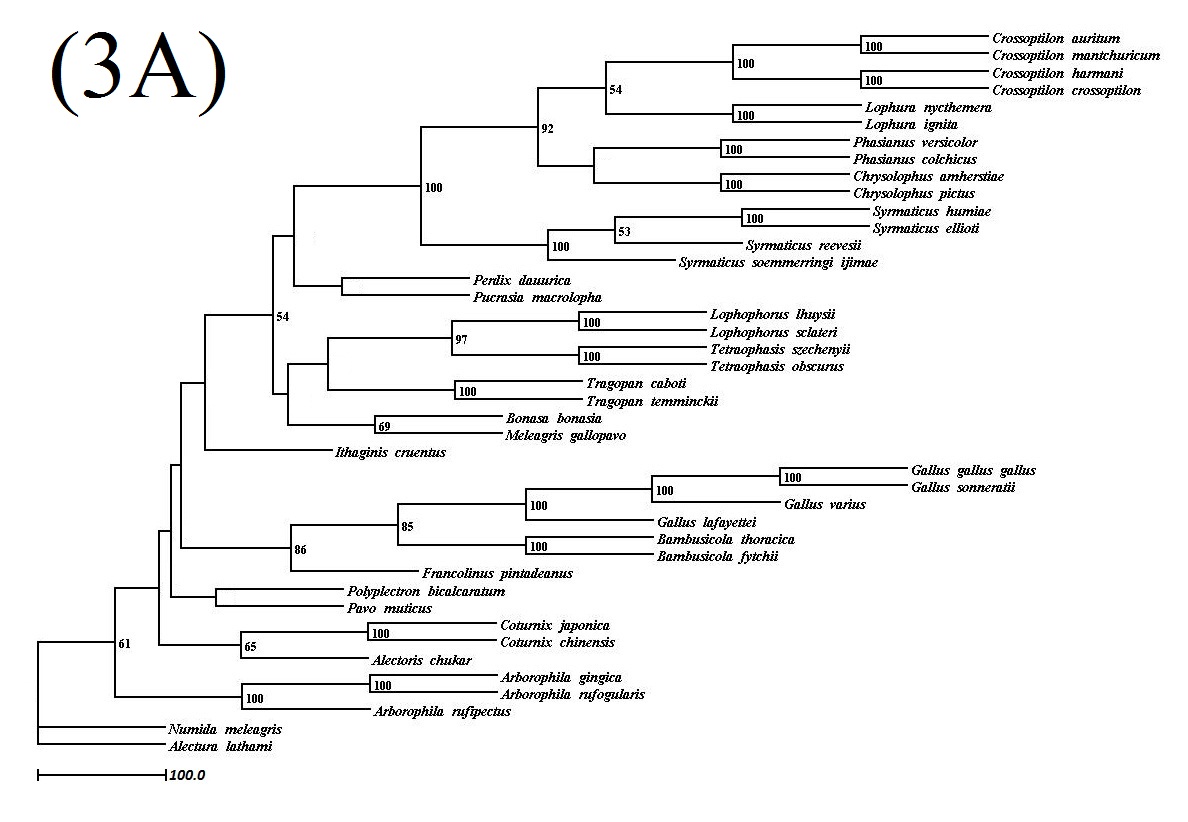


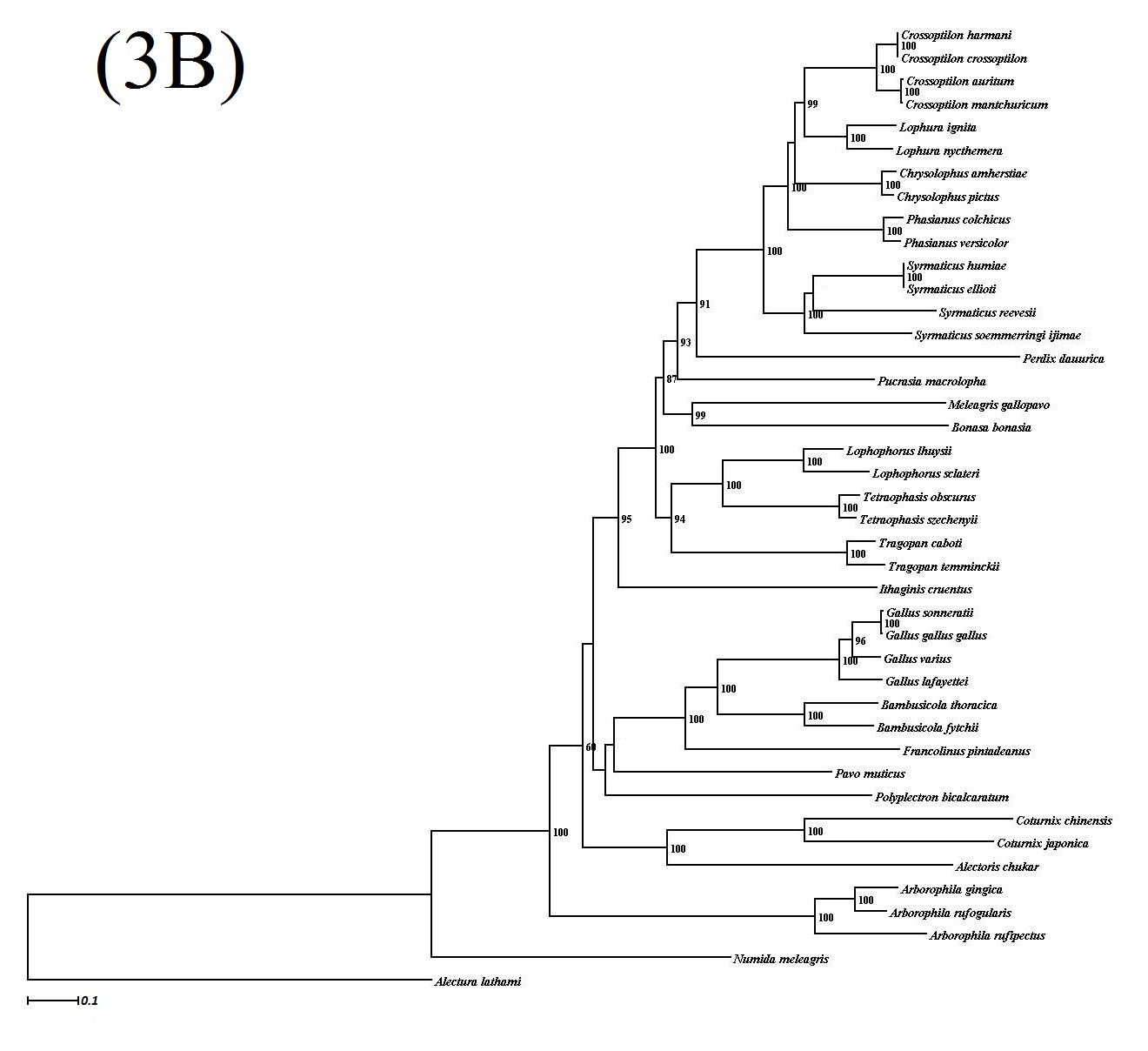


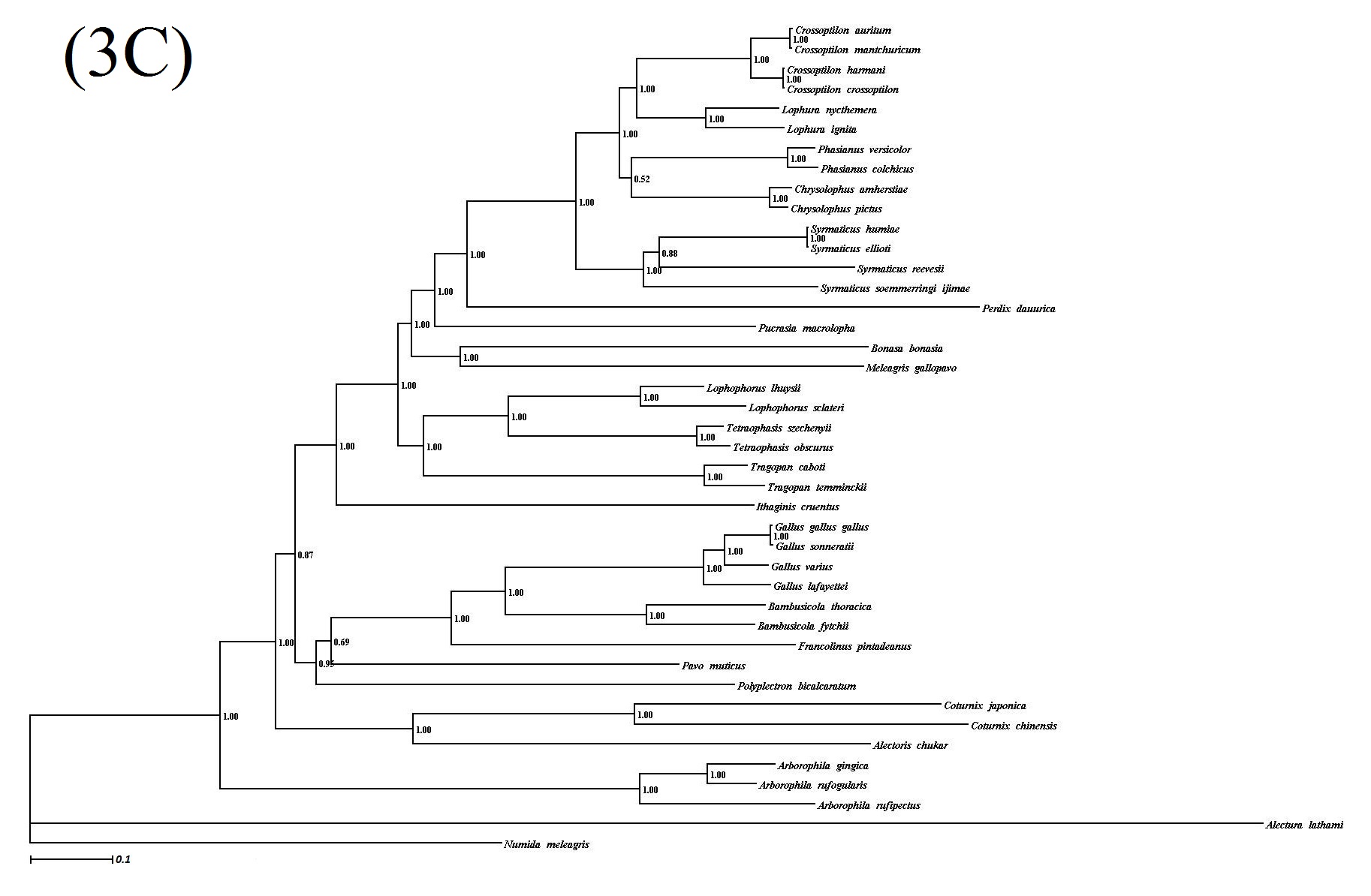


Additional file 8 - The MP, ML and BI trees of different datasets.

Less than 50% bootstrap values were omitted. Notes: (1) results based on PCG dataset; (2) results based on mitogenome dataset; (3) results based on the third codons dataset (3rd). (A) MP tree; (B) ML tree; (C) BI tree.
