# Supplementary material for: Comparative mitochondrial genomics and phylogenetic relationships of the Crossoptilon species (Phasianidae, Galliformes)
Source: BMC Genomics. 2015 Feb 5;16(1):42. doi: 10.1186/s12864-015-1234-9 (PMC4326528; doi:10.1186/s12864-015-1234-9)
Supplement: Additional file 9: — Interspecific and intraspecific genetic distances of the Crossoptilon species based on mitogenome dataset and single genes. Note: C. harmani: C.har, C. mantchuricum: C. man, C. crossoptilon: C. cro, C. auritum: C. aur. [file 12864_2015_1234_MOESM9_ESM.doc]

Additional file 9 - Interspecific and intraspecific genetic distances of the *Crossoptilon* species based on mitogenome dataset and single genes.

| Gene | inter-species | | | | | | intra-species | | | |
| --- | --- | --- | --- | --- | --- | --- | --- | --- | --- | --- |
|  | *C.aur-*  *C.har* | *C.aur*-  *C.man* | *C.aur*-  *C.cro* | *C.har*-  *C.man* | *C.har*-  *C.cro* | *C.man-*  *C.cro* | *C.aur* | *C.cro* | *C.har* | *C.man* |
| mitogenomes | 0.026 | 0.002 | 0.026 | 0.026 | 0.001 | 0.026 |  |  |  |  |
| *nad2* | 0.03 | 0.001 | 0.029 | 0.03 | 0.001 | 0.03 | 0.001 | 0.001 | 0 | 0.001 |
| *Cytb* | 0.02 | 0.001 | 0.025 | 0.02 | 0.006 | 0.025 | 0.001 | 0.002 | 0.01 | 0.001 |
| *rrnS* | 0.016 | 0.002 | 0.016 | 0.016 | 0.001 | 0.016 | 0 | 0.001 | 0.001 | 0.002 |
| CR | 0.038 | 0.002 | 0.036 | 0.039 | 0.003 | 0.038 | 0.001 | 0.002 | 0.006 | 0.002 |

Note: *C. harmani*: *C.har*, *C. mantchuricum*: *C. man*, *C. crossoptilon*: *C. cro*, *C. auritum*: *C. aur*
