# Supplementary material for: Comparative mitochondrial genomics and phylogenetic relationships of the Crossoptilon species (Phasianidae, Galliformes)
Source: BMC Genomics. 2015 Feb 5;16(1):42. doi: 10.1186/s12864-015-1234-9 (PMC4326528; doi:10.1186/s12864-015-1234-9)
Supplement: Additional file 10: — The variable sites of protein-coding genes among six Crossoptilon groups. Note: C. harmani: C.har, C. mantchuricum: C. man, C. crossoptilon: C. cro, C. auritum: C. aur. [file 12864_2015_1234_MOESM10_ESM.doc]

Additional file 10 - The variable sites of protein-coding genes among six *Crossoptilon* groups.

| Gene | Length | Groups | | | | | |
| --- | --- | --- | --- | --- | --- | --- | --- |
|  |  | *C.har*-*C.man* | *C.man*-*C.cro* | *C.har*-*C.cro* | *C.har*-*C.aur* | *C.man*-*C.aur* | *C.cro*-*C.aur* |
| *nad1* | 972 | 38 | 38 | 0 | 35 | 4 | 35 |
| *nad2* | 1038 | 32 | 32 | 0 | 33 | 1 | 33 |
| *cox1* | 1548 | 47 | 48 | 1 | 48 | 3 | 49 |
| *cox2* | 681 | 14 | 14 | 0 | 16 | 2 | 16 |
| *atp8* | 162 | 3 | 3 | 0 | 4 | 1 | 4 |
| *atp6* | 681 | 14 | 13 | 1 | 13 | 1 | 12 |
| *cox3* | 783 | 27 | 26 | 1 | 29 | 2 | 28 |
| *nad3* | 348 | 13 | 16 | 3 | 14 | 1 | 17 |
| *nad4L* | 294 | 7 | 7 | 0 | 7 | 0 | 7 |
| *nad4* | 1377 | 48 | 48 | 0 | 47 | 5 | 47 |
| *nad5* | 1815 | 60 | 55 | 5 | 60 | 2 | 55 |
| *cytb* | 1140 | 28 | 27 | 1 | 28 | 0 | 27 |
| *nad6* | 519 | 24 | 24 | 0 | 24 | 0 | 24 |

Note: *C. harmani*: *C.har*, *C. mantchuricum*: *C. man*, *C. crossoptilon*: *C. cro*, *C. auritum*: *C. aur*
